# The impact of unconditional child cash grant on child malnutrition and its immediate and underlying causes in five districts of the Karnali Zone, Nepal – A trend analysis

**DOI:** 10.1186/s13690-019-0352-2

**Published:** 2019-05-29

**Authors:** Andre M. N. Renzaho, Wen Chen, Sanjay Rijal, Pradiumna Dahal, Ingrid R. Chikazaza, Thakur Dhakal, Stanley Chitekwe

**Affiliations:** 1 School of Social Sciences and Psychology, Western Sydney University, Locked Bag 1797, Penrith, NSW 2751 Australia; 2 Faculty of Medical Statistics and Epidemiology, School of Public Health, Sun Yat-sen University, Guangzhou, 510080 China; 3 UNICEF Nepal, UU House Pulchowk, Po Box 1187, Kathmandu, Nepal; 4 Independent consultant, Kathmandu, Nepal

**Keywords:** social protection, child cash grant, Nepal, IYCF, WASH, malnutrition

## Abstract

**Background:**

The impact of unconditional cash transfers on child malnutrition and its determinants remains poorly understood. The aim of this study was evaluate the impact of an unconditional child cash grant on children’s nutritional status and its immediate (infant and young child feeding, dietary diversity, food consumption, and child infection and care) and underlying (household food security; Water, Hygiene and Sanitation (WASH) determinants among children younger than five years in the Karnali Zone, Nepal.

**Methods:**

The five districts of the Karnali Zone received standard social welfare services in the form of targeted resource transfers for eligible families, plus an unconditional child cash payment, augmented by a capacity building and behavioural change education. Repeated cross-sectional surveys, with measures taken at baseline (2009, *N*=3750), midline (2013, *N*=3750) and endline (2015, *N*=3647), were carried out using a two-stage cluster sampling method. Multi-level Generalized Linear Mixed Models (GLMMs) with normal, binomial, Poisson, or multinomial link were performed to detect the unadjusted and adjusted trends.

**Results:**

There was a linear growth among children, with a corresponding increase of 0.41 height-for-age Z-scores (p < 0.001), 0.50 weight-for-age Z-scores (*p*<0.001), and 0.34 weight-for-height Z-scores (*p*<0.001) between the study period, equating to a decline in child undernutrition of 9.4, 16.5, and 5.1 percentage points (*p*<0.001) for stunting, underweight, and wasting respectively. Improvements were also observed in WASH outcomes, care and health seeking behaviours, and food availability.

**Conclusion:**

Unconditional child cash grant embedded within a government sponsored cash transfer program for families and complemented by capacity building and behavioural change strategies improves child nutritional status and its determinants.

**Electronic supplementary material:**

The online version of this article (10.1186/s13690-019-0352-2) contains supplementary material, which is available to authorized users.

## Introduction

Despite improvements over the last two decades, addressing child malnutrition in low and middle income countries remains a challenge. The United Nations Children's Fund (UNICEF) conceptual framework provides the best opportunity to better understand causes of child malnutrition [1, 2]. It conceptualises multiple causes of child malnutrition as immediate (individual level), underlying (household/community level) and basic (societal level). The immediate causes include inadequate dietary intake and infection, and the interaction between them. That is, reduced appetite associated with an infection makes it impossible for the child who has an infection to meet their increased nutrient requirements; while poor nutrition reduces immunity and makes the body more susceptible to infection. The underlying causes are more concerned with three dimensions and their interaction: 1) food security (food availability, access, utilisation, and asset creation); 2) inadequate child care practices; and 3) an impaired public health environment such as poor water and sanitation and inadequate health services. Underlying causes are however, influenced by societal level basic structural factors such as the political, economic, cultural, religious, and institutional structures that govern society [2].

Both Conditional Cash Transfer (CCT) and Unconditional Cash Transfer (UCT) programs have emerged as a powerful instrument to improve child health, but results have varied greatly [3, 4]. The strongest evidence comes from CCT and its overall impact on child malnutrition. Factors associated with the effectiveness of cash transfer programs in addressing child malnutrition have included compliance with nutritional monitoring and advice to mothers about child nutrition, giving the cash to women rather than men, targeting older women (>=50 years) over younger women, transfer size (higher cash transfer levels are associated with improvements in effect than relatively small cash transfer), duration of exposure (greater exposure to regular cash transfers), conditionalities (conditional on regularly or quarterly visits to health centres for child growth monitoring and treatment), payment mechanism (mobile payment having a greater effect than manual payment), and complementary interventions and supply-side services (especially complementary nutritional supplements in addition to cash transfers led to significantly greater reductions than cash only) [3–5].

Studies examining the effect of cash transfers on the immediate and underlying causes of child malnutrition have been limited and varied. The few that exist focus on dietary intake, food security, and maternal child health with mixed results [3, 6]. For example, of the 12 studies reporting the overall cash transfer effect on dietary diversity measures in the Bastagli et al’s study [3], seven of them found at least one statistically significant improvement in dietary diversity. Cash transfers have also been found to increase household food security and food diversity [3, 6], and the use of maternal and child health facilities (increased antenatal visits, attendance at growth monitoring centres and health check-ups, skilled attendance at birth, delivery at a health facility, tetanus toxoid vaccination for mothers and the reduced incidence of low birthweight) [7, 8].

There exist significant gaps in the study of cash transfers and their impact on critical underlying and immediate causes of malnutrition. Despite the interaction between infection and dietary intake, studies examining the impact of cash transfers on child infection are rare. The few limited studies that exit suggest that cash transfers can have an impact on child health and infections, including lowering respiratory tract infections in children [9], reducing HIV and HSV-2 infections in adolescent schoolgirls [10], and decreasing childhood mortality overall [11]. While water, sanitation and hygiene (WASH) factors are important contributors to children’s nutrition outcomes, there is no robust evidence of cash transfer programs’ impact on WASH outcomes [12]. The hypothesis is that social protection programs may improve WASH outcomes by removing social and financial barriers and affecting behavioural changes, but this hypothesis has only been limited to few studies and remains fully untested in non-emergency settings [12]. Finally, most of the studies on cash transfers have been implemented in isolation and/or as randomised controlled trials. While randomised controlled trials increase the internal validity, they tend to suffer from external validity because of the huge difference between the trial protocol and routine practice [13].

Therefore, the aim of this study was to evaluate the impact of an unconditional child cash grant on children’s nutritional status and its immediate and underlying causes in five districts of the Karnali Zone (Kalikot, Jumla, Mugu, Humla and Dolpa districts), Nepal. The child cash grant was embedded within a government sponsored cash transfer program for families and complemented by capacity building and behavioural change education. By implementing the program through real-life settings, the project sought to maximise the ecological validity of the findings.

## Methodology

### The intervention

Prior to the introduction of the unconditional child cash grants, families in the Karnali Zone received government-funded Targeted Resource Transfers (TRTs) that consisted of: senior citizens allowance for all persons aged 70+ (500 Nepalese rupees [NRs]/month; 1USD=NRs103), single women and widow allowance (NRs 500/month), disability allowance for all people with disability aged 16 years or older (NRs 1000/month for total disability and NRs 300/month for partial disability), endangered ethnicities allowance (all household members receive NRs 500/month), and maternity incentive scheme for pregnant women (NRs 500 in Tarai, NRs 1000 in Hills and NRs 1500 in mountains as transportation costs plus NRs 300 provided to health professionals and NRs 1000 reimbursement to facilities plus free delivery care).

In the Government of Nepal’s (GoN’s) 2009/2010 budget, the TRTs were augmented with an unconditional child cash grant (CCG) program. The CCG provides NRs 200 per month for up to two children for families with children under five. The CCG has been enhanced by a capacity building for social protection, implemented by a United Nations Children’s Fund (UNICEF)/Nepal partnership program, with the financial support from the Asian Development Bank and the Japan Fund for Poverty Reduction (Additional file 1: Table S1). UNICEF has been responsible for the implementation of the capacity development and linking CCG with nutrition and supporting the GoN (Ministry of Federal Affairs and Local Development and Ministry of Health and Population) in implementing key strategies underpinning the intervention. These are:

1. Capacity building to enhance the capacity of local bodies in the project districts to deliver the child grant, through orientations for Village Development Committee (VDC) leaders, Traditional Healers and mothers/caretakers, and capacity-building for health workers and Female Community Health Volunteers (FCHVs) and VDC secretaries;
2. Enhancing networking between local bodies, health facilities and communities in the project districts to improve child nutrition;
3. Social behaviour change communication on child nutrition including the provision of nutrition-related counselling services;
4. Awareness raising for timely birth registration, to identify all eligible households and inform them about the availability of the CCG;
5. Assisting mothers and others caring for children to identify the best possible locally available food and encouraging them to use the CCG for nutritious foods and the improvement of the nutritional status of children; and
6. Improving the knowledge and skills of CCG beneficiaries in the areas of Infant and Young Child Feeding (IYCF) practices, hygiene, sanitation, and other key behaviours linked to child nutrition.

### Study design and sampling strategy

**Fig. 1:**
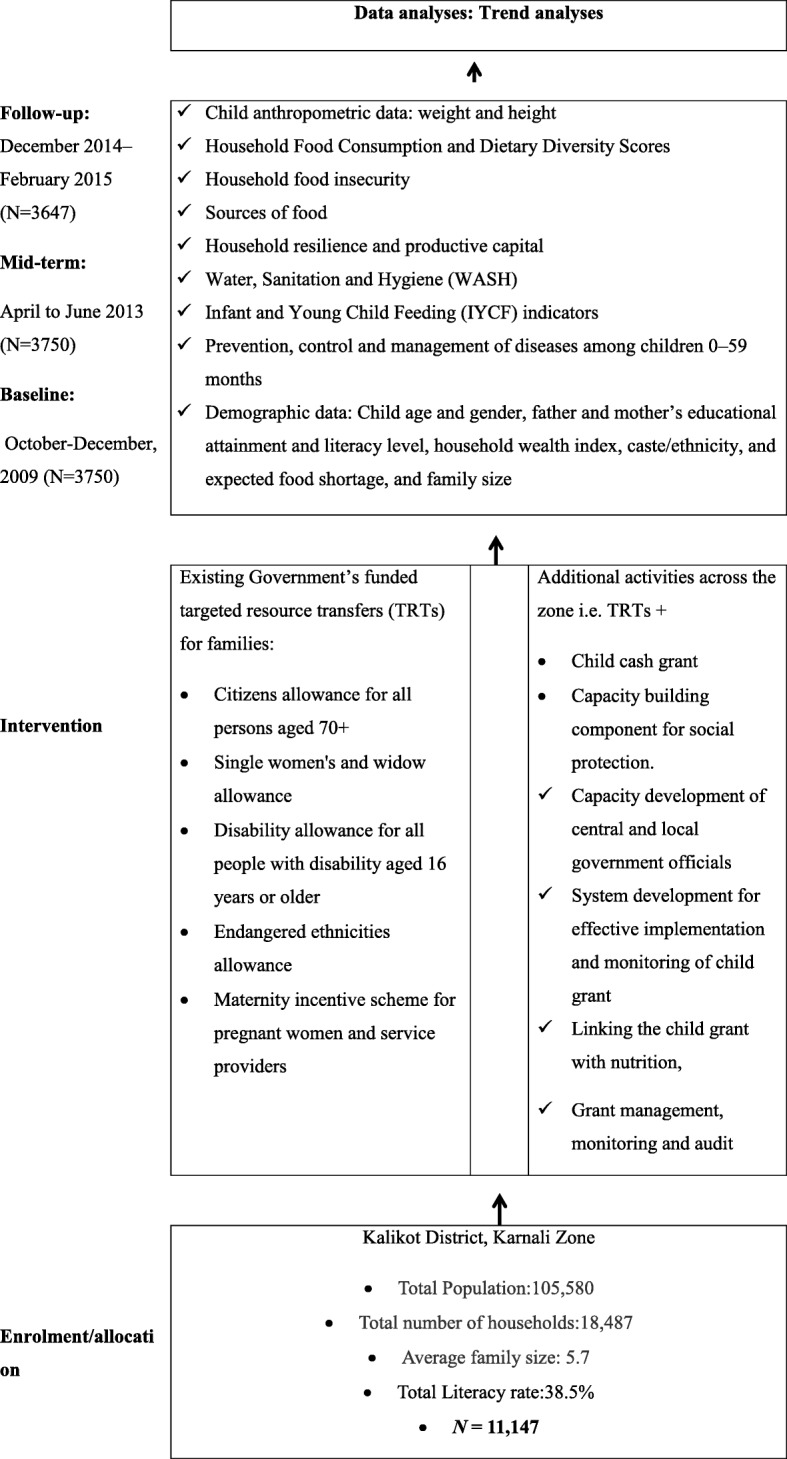
Flow diagram detailing the intervention implementation plan and data collection phases

The study carried repeated cross-sectional surveys, with measures taken pre- (October–December 2009, N=3750), mid- (April to June 2013, N=3750), and post- (December 2014–February 2015, N=3647) intervention in the five districts of the Karnali Zone. The whole design is summarised in Fig. 1. The study was approved by the Nepal Health Research Council Ethical Review Board (approval no. 2071-12-18; Reg No. 29/2015).

Households to be surveyed were selected using a two-stage cluster sampling method. The sampling approach has been described elsewhere [14]. Briefly, the first stage involved identifying clusters (wards) within each district to be included in the study. All wards in each district were listed separately in alphabetical order by VDC. Using the 2011 population census data for each ward (cluster), a cumulative population for all wards was computed. From this cumulative list, the required number of clusters in each district was determined using the probability proportional to size sampling method.

The second stage involved selecting households within retained clusters. A list of households in each selected cluster (ward) was constructed with the help of the local leaders and UNICEF staff. From the list, a household was selected using a systematic sampling approach. Only households with at least one child under 60 months of age were eligible for the study. The sampling interval (X) was determined by dividing the total number of households in each cluster (ward) with the expected sample size, and the first household to be surveyed was randomly selected by choosing a number between 1 and X. For each selected household, mothers/caretakers of children under five years of age volunteered to take part in the surveys, and the interview occurred outside the home, away from other household members. If the selected household was not inhabited, or there was no one at home, the closest neighbouring household was used for the survey. We sampled approximately 30 households per cluster in each district at each measurement point. For clusters where the number of households was less than 25, the selected ward and its adjoining neighbour were merged and treated as a single cluster. In households with more than one child, only one child was randomly selected for enumeration.

## Study variables

### Dependent variables

#### Child anthropometric data

Outcome variables were anthropometric indices, namely Height-for-age Z-scores (HAZ), Weight-for-age Z-scores (WAZ), and Weight-for-height Z-scores (WHZ). Weight and height data were collected by trained enumerators. Weight was measured using a SECA (Hamburg, Germany) digital scale to the nearest 0.1 kg. Height was measured using a measuring board made by Shorr Productions for use in survey settings to the nearest 0.1 cm. Children below two years of age were measured in supine position (lying down) while those over two years were measured standing up. HAZ, WAZ, and WHZ were generated using the 2006 World Health Organization (WHO) [15] Child Growth Standards and three types of child malnutrition were considered in this study: wasting (WHZ below minus two standard deviations and/or bilateral oedema), stunting (HAZ below minus two standard deviations), and underweight (WAZ below minus two standard deviations). To increase the accuracy of the anthropometric indices, implausible values were excluded. Biologically implausible values were defined using the WHO standards-based results as follows: z-scores of <−5 or > +5 for WHZ; <−6 or >+6 for HAZ; and <-6 or >+5 for WHZ [15].

#### Immediate cause of malnutrition

Immediate cause of child malnutrition considered two indicators. The first was *the prevention, control and management of diseases among children 0–59 months*, which focused on the integrated community case management and community-based health planning and services for fever, diarrhoea, and pneumonia. Indicators included action taken in response to child illness, such as seeking advice, giving more fluid and food and continuing breastfeeding [16, 17]. In terms of prevention, we looked at Vitamin A supplementation, deworming, the use of iodised salt, and immunisation status, with a particular emphasis on immunisation against DPT and measles.

The second immediate cause of child malnutrition focused on Household Food Consumption Scores (FCS) and Household Dietary Diversity Scores (HDDS). The food consumption scores (FCS) represent a composite score that takes into account dietary diversity, food frequency and relative nutrition importance of different food groups. Therefore, Nepal-specific food items were used to compute the FCS according to the World Food Program’s food consumption analysis module [18]; and the sum of all the consumption frequencies of food items of the same group were used during the seven days before the survey to create food group scores. Any value of each food group above 7 was recoded as 7. The obtained score for each food group was multiplied by its weight as recommended by the World Food Program [18]. The food group scores used for the FCS were expanded from 9 to 11 food groups (expanding the meat and fish food group into discrete meat, fish and egg food groups). The food groups were then transformed into dichotomous variables such that 1 (Yes) represented household consumption of the specific food group and 0 (No) households that did not consume that food group. The dichotomous variables were summed to obtain a HDDS, with the score ranging from 0 through to a maximum of 11. The HDDS was classified into the following categories using the following cut-off points: 6+: high (good) dietary diversity; 4.5–6: medium dietary diversity, and <4.5: low dietary diversity.

### Underlying causes of child malnutrition

The first underlying cause of child malnutrition was food insecurity. Household Food Insecurity Access Scale (HFIAS) [19], a brief nine-item instrument used to assess problems experienced by households in accessing food during the last 30 days preceding the survey was used. The frequency of experienced problems during the last 30 days was recorded on a three-point scale: rarely (1–2 times), sometimes (3–10 times), or often (more than 10 times). Each of the nine questions had a possible score of 0–3, for a possible total score ranging from 0 to 27 (with higher scores an indication of food insecurity). The score was classified into tertiles, with the lowest tertile being ‘food secure’, the middle tertile equating to ‘moderately food insecure’ and the highest tertile corresponding to ‘severely food insecure.

Sources of food and household resilience and productive capital were measured. Main sources of food considered included food obtained through own production, purchasing, borrowing, gifts, hunting and food aid. Household resilience was conceptualized as the ability to manage and withstand shocks in difficult circumstances. Indicators considered were temporary out-migration as a coping strategy in times of difficulty (e.g. circular migration between home and host areas for the purpose of employment) and financial security, including being out of debt (e.g. not borrowing money for survival or out-migration to look for money to pay off the debt), having enough food to eat (e.g. not experiencing food shortage), and having diversified sources of income. Productive capital included already-produced, durable non-financial assets used in production of goods or services, including ownership of assets, land, and agricultural assets and inputs. The indicator was the preservation of these assets.

The second underlying cause of child malnutrition included *WASH outcomes*, generated according to the World Health Organisations’ guidelines [20]: ***improved sources of drinking-water*** (piped water into dwelling, piped water to yard/plot, public tap or standpipe, tube well or borehole, protected dug well, protected spring, bottled water, and rainwater) vs. ***unimproved sources of drinking-water*** (unprotected spring, unprotected dug well, cart with small tank/drum, tanker-truck, surface water); ***adequate water treatment methods*** (boiling; adding bleach/chlorine; using a water filter such as ceramic, sand, composite, and solar disinfection) vs. ***inadequate water treatment methods*** (straining it through a cloth or letting it stand and settle); . ***improved sanitation facilities*** (flush toilet, piped sewer system, a septic tank, a flush/pour flush to pit latrine, a ventilated improved pit, a pit latrine with slab, and a composting toilet) vs. ***unimproved sanitation facilities*** (a flush/pour flush to elsewhere, a pit latrine without slab, bucket/containers, a hanging toilet or hanging latrine, bush/ field or no facilities); and . ***sanitary disposal of children’s faeces*** (child used toilet/latrine, put/rinsed faeces into the toilet or latrine or buried the faeces) vs. ***unsanitary disposal of children’s faeces*** (put/rinsed faeces into drain or ditch, faeces thrown into the garbage or faeces left or buried in the open).

The last underlying cause of child malnutrition included in the study focused on *IYCF* indicators. IYCF indicators followed the World Health Organization [21] prescribed indicators; these included: ever breastfed, early initiation of breastfeeding, exclusive breastfeeding under 6 months, exclusive breastfeeding for infants 4–5 months, continued breastfeeding at 1 year, continued breastfeeding at 2 years, bottle feeding, introduction of solid, semi-solid or soft foods, minimum dietary diversity, minimum meal frequency, minimum acceptable diet, consumption of iron-rich or iron-fortified foods the previous day, consumption of Vitamin A-rich foods the previous day, and consumption of protein foods the previous day. The operational definitions of these indicators are summarised in Additional file 1: Table S2.

#### Independent variables

The time of measurement was considered in all analyses to depict the time trend and adjust all the regression models for time-dependent confounders. Additional individual and household factors were assessed using a structured questionnaire and included questions concerning: mother and father literacy (0=no, 1=yes) and educational attainment (0=None, 1=Primary Level, 2=Lower Secondary Level, 3=secondary Level, 4=Higher Secondary, and 5=Intermediate and Above), child age (in months) and gender (0=girls, 1=boys), and harvest failure (0=no, 1=yes) and Household Wealth Index (HWI). The HWI was computed according to the Demographic and Health Survey’s module [22]. It was a composite measure of a household’s cumulative living standard and was generated using the principal component analysis to produce the relative economic status of households based on an analysis ownership of selected assets, including televisions and bicycles; materials used for housing construction (e.g. the type of floor, wall, and roof materials); members per sleeping room; agricultural land (e.g. ownership of agricultural land and the amount of land owned); farm animals/livestock (e.g. ownership of farm animals and the numbers of different types of animals); and the types of water access and sanitation.

### Data analysis

Analyses were conducted using IBM SPSS Statistics 21.0 (IBM Corp. Armonk, NY). Analyses of the time trend in the prevalence of dependent variables from 2009 to 2015 were carried out. Bivariate multi-level Generalized Linear Mixed Models (GLMMs) with normal, binomial, Poisson, or multinomial link were performed to detect the unadjusted time trend while only adjusting for clustering effects. We did not include the household level error term because of the data structure (only one child under five was surveyed in each household). Normal link was used for HAZ, WAZ, and WHZ; binomial link was used for stunting, underweight, and wasting; binomial or Poisson link was used for WASH, IYCF, child disease prevention and management, sources of staple foods, resilience indicators, reproductive capital indicators and source of income; and multinomial link was used for household food consumption, dietary diversity, and food security (Table 1). Poisson link was used for variables where the prevalence was low and the sample size was large, because probabilities from the Poisson distribution approximate probabilities from the binomial distribution [23]. In the trend analyses of data in the Karnali Zone as a whole, three-level GLMMs were performed, namely the study children clustered into households, households clustered into wards, and wards clustered into districts. Similarly, two-level GLMMs were carried out for trend analyses on data in each district (children clustered into households, and households clustered into wards). In each district, two-level multivariate GLMMs with normal, binomial, Poisson, or multinomial link were performed to estimate adjusted effects of time (midline vs. baseline, and endline vs. baseline) after controlling for a set of control variables in every regression, notably child age and gender, father and mother’s educational attainment, household wealth index, caste/ethnicity, family size, and clustering effects. Multicollinearities among independent variables were tested by using Variance Inflation Factor (VIF). VIFs of all independent variables were between 1 and 1.5, which indicated there was no multicollinearity [24]. The goodness of fit of the models was assessed by the Bayesian information criterion (BIC). We compared the BIC of unadjusted and adjusted models. The adjusted model had lower BIV values, therefore, retained in the final analyses [25]. Dependent on the distribution of each key variable, adjusted linear regression coefficient of time (A*β* for normal distribution) or adjusted odds ratio of time (AOR for binomial, Poisson, or multinomial distribution), and 95% confidence interval (CI) were calculated. In all cases *P*-value < 0.05 was considered to be statistically significant.

**Table 1:** Dependent variables and link functions used in Generalized Linear Mixed Models

Variable	Type	Link function
Stunting	Binary	Logit
Underweight	Binary	Logit
Wasting	Binary	Logit
HAZ	Continous	Normal
WAZ	Continous	Normal
WHZ	Continous	Normal
‘Unimproved’ drinking water sources	Binary	Logit
‘Inadequate’ water treatment method	Binary	Logit
‘Unimproved’ sanitation facilities	Binary	Logit
‘Unsanitary’ disposal of children’s faeces	Binary	Logit
Early initiation of breastfeeding	Binary	Logit
Bottle feeding	Binary	Logit
Diarrhoea	Binary	Logit
Pneumonia	Binary	Logit
Fever	Binary	Logit
Sought advice	Binary	Logit
More fluid	Binary	Logit
More food#	Binary	Poisson
More breastfeeding	Binary	Logit
Deworming	Binary	Logit
Iodised salt	Binary	Logit
Household food consumption	Multinomial	Logit
Household dietary diversity	Multinomial	Logit
Household food security	Multinomial	Logit
Own production	Binary	Logit
Purchasing	Binary	Logit
Food aid	Binary	Logit
Outmigration	Binary	Poisson
Borrowing money	Binary	Logit
Selling land	Binary	Poisson
Selling agricultural assets	Binary	Poisson
Crop farming	Binary	Logit
Livestock farming	Binary	Poisson
Employment	Binary	Logit

## Results

### Child nutritional status

**Fig. 2:**
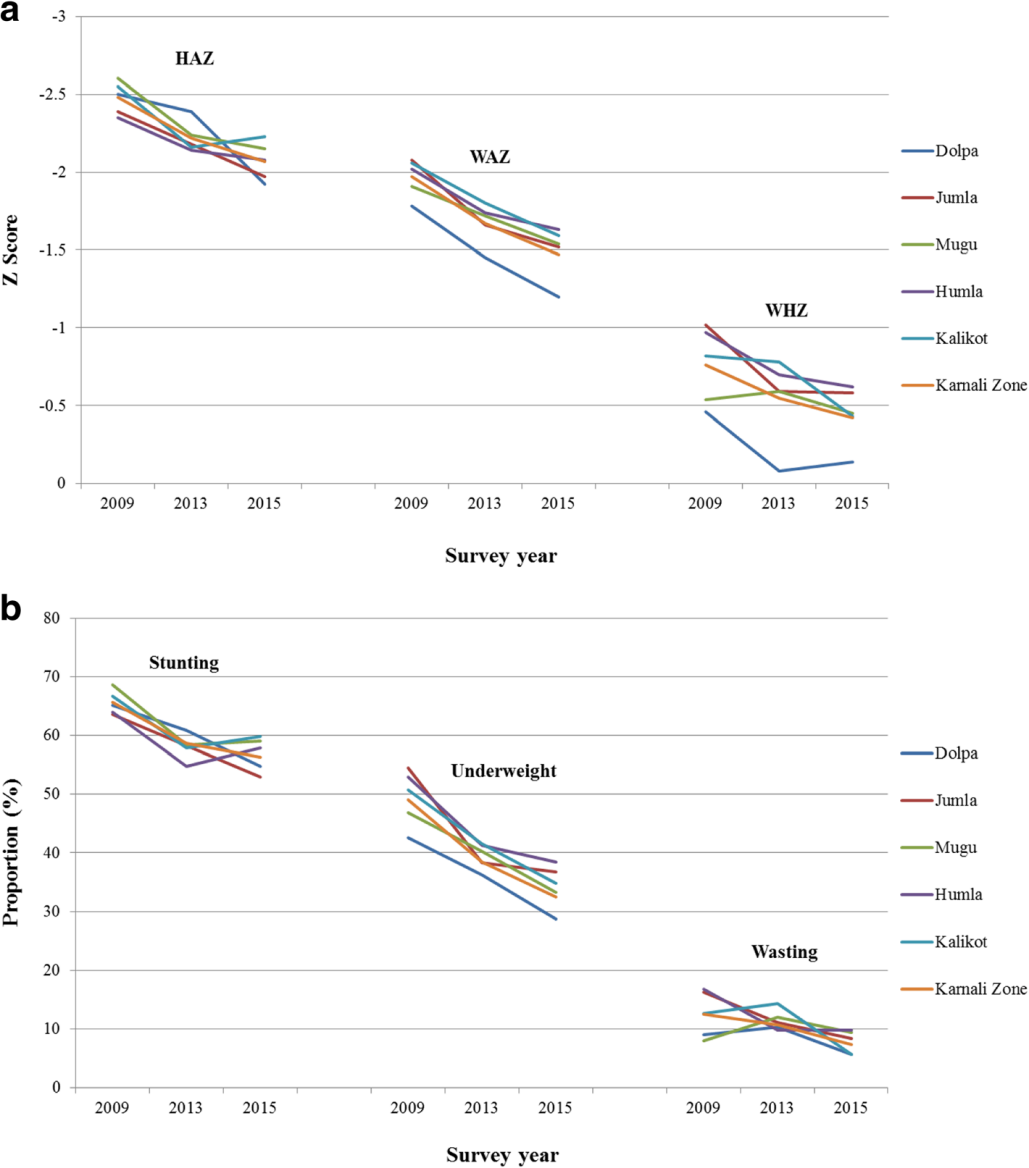
**a** Trend in HAZ, WAZ, and WHZ among children under five in the Karnali Zone of Nepal, 2009-2015. **b**: Trend in the prevalence of stunting, underweight, and wasting among children under five in the Karnali Zone of Nepal, 2009-2015

The demographic characteristics of the study participants are summarised in Tables 2 and 3. Across the Karnali Zone, there was a linear growth among children, with a corresponding increase of 0.41 HAZ (*p* < 0.001), 0.50 WAZ (*p*<0.001), and 0.34 WHZ (*p*<0.001) between the study period i.e. between 2009 and 2015 (Fig. 2a, Additional file 1: Table S3A), corresponding to a decline in child malnutrition of 9.4, 16.5, and 5.1 percentage points (*p*<0.001) for stunting, underweight, and wasting respectively (Fig. 2b, Additional file 1: Table S3B). Analyses by districts suggest there was a downward trend in the prevalence of child underweight and stunting across all districts, and child wasting for Jumla, Humla, and Kalikot districts. The results remained consistent after adjusting for socio-demographic and economic factors, except for Humla, Jumla, and Kalikot districts, where the trend for stunting became non-significant (Table 4).

**Table 2:** Characteristic of participants at baseline and follow-up in the Karnali Zone of Nepal, 2009-2015

Variables	Baseline (2009)		Midline (2013)		Endline (2015)		*P-value*
	*N*	*%*	*N*	*%*	*N*	*%*	
No. of households surveyed	3750	100	3750	100	3647	100	
No. of people per household [Mean(SD)]	3750	6.5(2.4)	3750	6.4(2.4)	3647	6.1(2.3)	<0.001
Child's age in months [Mean (SD)]	3750	28.5(15.4)	3750	28.0(15.6)	3647	28.2(15.5)	0.378
0-5	274	7.3	313	8.3	255	7.0	0.649
6-11	378	10.1	385	10.3	399	10.9	
12-23	837	22.3	813	21.7	814	22.3	
24-35	943	25.1	921	24.6	908	24.9	
36-47	813	21.7	816	21.8	769	21.1	
48-60	505	13.5	502	13.4	502	13.8	
Children’s gender							0.681
Girls	2000	53.3	1963	52.3	1934	53.0	
Boys	1750	46.7	1787	47.7	1713	47.0	
Father’s education							<0.001
Illiterate	1751	46.7	1422	37.9	1173	32.2	
Primary Level	36	1.0	679	18.1	694	19.0	
Lower Secondary Level	612	16.3	255	6.8	237	6.5	
Secondary Level	896	23.9	473	12.6	439	12.0	
Higher Secondary	352	9.4	366	9.8	491	13.5	
Intermediate And Above	103	2.7	55	14.8	613	16.8	
Mother’s education							<0.001
Illiterate	3019	80.5	2977	79.4	2606	71.5	
Primary Level	185	4.9	316	8.4	350	9.6	
Lower Secondary Level	266	7.1	71	1.9	100	2.7	
Secondary Level	192	5.1	167	4.5	218	6.0	
Higher Secondary	77	2.1	115	3.1	178	4.9	
Intermediate And Above	11	0.3	104	2.8	195	5.3	
Ethnicity							<0.001
Dalit Hill/Terai	770	20.5	878	23.4	778	21.3	
Disadvantage Janajati/Hill/Terai/Non Dalit Terai	464	12.4	259	6.9	214	5.9	
Relatively Advantaged Janajati	28	0.7	130	3.5	119	3.3	
Upper caste Group	2488	66.3	2483	66.2	2536	69.5	
Household wealth index							
Poorest	1335	37.9	352	10.2	182	5.4	<0.001
Poorer	1030	29.3	592	17.1	577	17.1	
Middle	548	15.6	723	20.9	937	27.7	
Richer	446	12.7	833	24.1	784	23.2	
Richest	161	4.6	952	27.6	897	26.6	
Expected food shortage for the coming months							
No	1384	36.9	1004	26.8	1199	32.9	<0.001
Yes	2366	63.1	2746	73.2	2448	67.1

**Table 3:** Characteristic of participants at baseline and follow-up in the five districts in Karnali Zone of Nepal, 2009-2015

Variables	Dolpa			Jumla			Mugu			Humla			Kalikot		
	2009	2013	2015	2009	2013	2015	2009	2013	2015	2009	2013	2015	2009	2013	2015
No. of households surveyed	750	750	947	750	750	575	750	750	750	750	750	625	750	750	750
No. of people per household [Mean(SD)]	6.1(2.1)	5.5(1.8)	5.7(1.9)	6.4(2.4)	6.6(2.5)	6.3(2.8)	6.7(2.4)	6.2(2.1)	6.1(2.2)	6.7(2.5)	6.5(2.5)	6.5(2.4)	6.9(2.4)	7.0(2.6)	6.3(2.4)
Child's age in months [Mean (SD)]	29.6(16.0)	29.4(15.3)	28.8(15.7)	27.7(14.7)	26.2(15.3)	26.0(15.6)	28.9(15.8)	27.0(15.7)	26.8(15.0)	27.4(14.9)	29.2(15.5)	30.7(15.2)	28.7(15.4)	28.0(16.2)	28.4(15.7)
0-5 *n(%)*	54(7.2)	49(6.5)	60(6.3)	61(8.1)	67(8.9)	56(9.7)	57(7.6)	72(9.6)	52(6.9)	52(6.9)	60(8.0)	33(5.3)	50(6.7)	65(8.7)	54(7.2)
6-11	75(10.0)	74(9.9)	108(11.4)	67(8.9)	90(12.0)	62(10.8)	71(9.5)	76(10.1)	94(12.5)	85(11.3)	63(8.4)	51(8.2)	80(10.7)	82(10.9)	84(11.2)
12-23	148(19.7)	134(17.9)	193(20.4)	168(22.4)	186(24.8)	156(27.1)	160(21.3)	178(23.7)	178(23.7)	176(23.5)	147(19.6)	123(19.7)	185(24.7)	168(22.4)	164(21.9)
24-35	181(24.1)	187(24.9)	234(24.7)	214(28.5)	179(23.9)	127(22.1)	199(26.5)	186(24.8)	198(26.4)	194(25.9)	190(25.3)	157(25.1)	155(20.7)	179(23.9)	192(25.6)
36-47	169(22.5)	192(25.6)	201(21.2)	155(20.7)	151(20.10	110(19.1)	146(19.5)	146(19.5)	144(19.2)	158(21.1)	182(24.3)	161(25.8)	185(24.7)	145(19.3)	153(20.4)
48-60	123(16.4)	114(15.2)	151(15.9)	85(11.3)	77(10.3)	64(11.1)	117(15.6)	92(12.3)	84(11.2)	85(11.3)	108(14.4)	100(16.0)	95(12.7)	111(14.8)	103(13.7)
Children’s gender *n(%)*															
Girls	395(52.7)	400(53.3)	449(47.4)	406(54.1)	409(5450	307(53.4)	387(51.6)	374(49.9)	415(55.3)	398(53.1)	365(48.7)	340(54.4)	414(55.2)	415(55.3)	423(56.4)
Boys	355(47.3)	350(46.7)	498(52.6)	344(45.9)	341(45.5)	268(46.6)	363(48.4)	376(50.1)	335(44.7)	352(46.9)	385(51.3)	285(45.6)	336(44.8)	335(44.7)	327(43.6)
Father's education *n(%)*															
Illiterate	347(46.3)	314(41.9)	363(38.3)	366(48.8)	252(33.6)	177(30.8)	375(50.0)	283(37.7)	199(26.5)	316(42.1)	303(40.4)	187(29.9)	347(46.3)	270(36.0)	247(32.9)
Primary Level	16(2.1)	155(20.7)	197(20.8)	0(0.00	134(17.9)	99(17.2)	4(0.5)	114(15.2)	125(16.7)	5(0.7)	130(17.3)	132(21.1)	11(1.5)	146(19.5)	141(18.8)
Lower Secondary Level	159(21.2)	53(7.1)	51(5.4)	95(12.7)	60(8.0)	42(7.3)	110(14.7)	47(6.3)	32(4.3)	120(16.0)	47(6.3)	43(6.9)	128(17.1)	48(6.4)	69(9.2)
Secondary Level	152(20.3)	84(11.2)	103(10.9)	184(24.5)	111(14.8)	59(10.3)	170(22.7)	108(14.4)	107(14.3)	193(25.7)	87(11.6)	70(11.2)	197(26.3)	83(11.1)	100(13.3)
Higher Secondary	60(8.0)	50(6.7)	98(10.3)	72(9.6)	78(10.4)	92(16.0)	69(9.2)	98(13.1)	144(19.2)	100(13.3)	88(11.7)	90(14.4)	51(6.8)	52(6.9)	67(8.9)
Intermediate And Above	16(2.1)	94(12.5)	135(14.3)	33(4.4)	115(15.3)	106(18.4)	22(2.9)	100(13.3)	143(19.1)	16(2.1)	95(12.7)	103(16.5)	16(2.1)	151(20.1)	126(16.8)
Mother's education *n(%)*															
Illiterate	543(72.4)	560(74.7)	692(73.1)	597(79.6)	576(76.8)	366(63.7)	649(86.5)	632(84.3)	520(69.3)	590(78.7)	643(85.7)	499(79.8)	640(85.3)	566(75.5)	529(70.5)
Primary Level	58(7.7)	92(12.3)	107(11.3)	26(3.5)	63(8.4)	58(10.1)	21(2.8)	44(5.9)	77(10.3)	50(6.7)	45(6.0)	44(7.0)	30(4.0)	72(9.6)	64(8.5)
Lower Secondary Level	77(10.3)	20(2.7)	30(3.2)	52(6.9)	13(1.7)	29(5.0)	49(6.5)	11(1.5)	12(1.6)	56(7.5)	11(1.5)	9(1.4)	32(4.3)	16(2.1)	20(2.7)
Secondary Level	58(7.7)	32(4.3)	50(5.3)	49(6.5)	50(6.7)	53(9.2)	23(3.1)	27(3.6)	47(6.3)	36(4.8)	25(3.3)	25(4.0)	26(3.5)	33(4.4)	43(5.7)
Higher Secondary	14(1.9)	22(2.9)	22(2.3)	22(2.9)	22(2.9)	38(6.6)	5(0.7)	23(3.1)	59(7.9)	18(2.4)	20(2.7)	28(4.5)	18(2.4)	28(3.7)	31(4.1)
Intermediate And Above	0(0.0)	24(3.2)	46(4.9)	4(0.5)	26(3.5)	31(5.4)	3(0.4)	13(1.7)	35(4.7)	0(0.0)	6(0.8)	20(3.2)	4(0.5)	35(4.7)	63(8.4)
Ethnicity *n(%)*															
Dalit Hill/Terai	163(21.7)	118(15.7)	189(20.0)	204(27.2)	120(16.0)	121(21.0)	123(16.4)	153(20.4)	172(22.9)	120(16.0)	173(23.1)	105(16.8)	160(21.3)	314(41.9)	191(25.5)
Disadvantage Janajati/Hill/Terai/Non Dalit Terai	278(37.1)	129(17.2)	138(14.6)	6(0.8)	7(0.9)	6(1.0)	114(15.2)	25(3.3)	23(3.1)	56(7.5)	94(12.5)	46(7.4)	10(1.3)	4(0.5)	1(0.1)
Relatively Advantaged Janajati	3(0.4)	129(17.2)	119(12.6)	2(0.3)	1(0.1)	0(0.0)	0(0.0)	0(0.0)	0(0.0)	22(2.9)	0(0.0)	0(0.0)	1(0.1)	0(0.0)	0(0.0)
Upper caste Group	306(40.8)	374(49.9)	501(52.9)	538(71.7)	622(82.9)	448(77.9)	513(68.4)	572(76.3)	555(74.0)	552(73.6)	483(64.4)	474(75.8)	579(77.2)	432(57.6)	558(74.4)
Household wealth index *n(%)*															
Poorest	89(12.8)	44(6.5)	42(4.8)	255(38.1)	30(4.6)	12(2.4)	343(47.8)	77(10.9)	0(0.0)	273(38.2)	137(19.4)	25(4.2)	375(51.8)	64(9.0)	103(14.5)
Poorer	161(23.2)	71(10.5)	194(22.0)	242(36.2)	44(6.7)	82(16.6)	210(29.2)	175(24.9)	9(1.3)	185(25.9)	188(26.6)	94(15.7)	232(32.0)	114(16.1)	198(27.9)
Middle	148(21.3)	80(11.9)	252(28.6)	117(17.5)	93(14.2)	197(40.0)	82(11.4)	213(30.3)	18(2.6)	118(16.5)	207(29.3)	232(38.8)	83(11.5)	130(18.3)	238(33.5)
Richer	187(26.9)	142(21.0)	216(24.5)	48(7.2)	154(23.4)	106(21.5)	67(9.3)	160(22.7)	175(25.2)	112(15.7)	144(20.4)	142(23.7)	32(4.4)	233(32.8)	145(20.4)
Richest	109(15.7)	338(50.1)	178(20.2)	7(1.0)	336(51.1)	96(19.5)	16(2.2)	79(11.2)	492(70.9)	27(3.8)	30(4.2)	105(17.6)	2(0.3)	169(23.8)	26(3.7)
Expected food shortage for the coming months *n(%)*															
No	326(43.5)	304(40.5)	528(55.8)	305(40.7)	185(24.7)	193(33.6)	344(45.9)	254(33.9)	271(36.1)	139(18.5)	70(9.3)	90(14.4)	270(36.0)	191(25.5)	117(15.6)
Yes	424(56.5)	446(59.5)	419(44.2)	445(59.3)	565(75.3)	382(66.4)	406(54.1)	496(66.1)	479(63.9)	611(81.5)	680(90.7)	535(85.6)	480(64.0)	559(74.5)	633(84.4)

**Table 4:** Trend in child undernutrition in the Karnali Zone of Nepal after controlling for confounders*, 2009-2015

Variables	2009 (Ref.)	Dolpa		Jumla		Mugu		Humla		Kalikot		Karnali Zone	
		AOR(95%CI)		AOR(95%CI)		AOR(95%CI)		AOR(95%CI)		AOR(95%CI)		AOR(95%CI)	
		2013	2015	2013	2015	2013	2015	2013	2015	2013	2015	2013	2015
Stunting^#^	1	0.82 (0.63-1.08)	0.54^***^ (0.43-0.69)	1.05 (0.75-1.46)	0.78 (0.59-1.05)	0.57^***^ (0.44-0.75)	0.53^**^ (0.36-0.79)	0.69^*^ (0.54-0.87) ^*^	0.78 (0.60-1.02)	0.90 (0.66-1.21)	0.90 (0.69-1.17)	0.75^***^ (0.66-0.84)	0.68^***^ (0.61-0.77)
Underweight^#^	1	0.75* (0.57-0.98)	0.50^***^ (0.38-0.64)	0.73 (0.53-1.01)	0.62^**^ (0.47-0.83)	0.73^*^ (0.57-0.94)	0.63^*^ (0.43-0.92)	0.63^***^ (0.50-0.80)	0.60^***^ (0.46-0.78)	0.71^*^ (0.54-0.95)	0.54^***^ (0.42-0.69)	0.71^***^ (0.63-0.80)	0.57^***^ (0.51-0.64)
Wasting^#^	1	0.82 (0.52-1.30)	0.66 (0.43-1.01)	0.58^*^ (0.35-0.95)	0.41^***^ (0.26-0.65)	1.49^*^ (1.01-2.21)	1.52 (0.84-2.76)	0.52^*^ (0.36-0.76) ^*^	0.54^**^ (0.36-0.82)	0.91 (0.59-1.39)	0.41^***^ (0.27-0.62)	0.77^**^ (0.65-0.93)	0.52^***^ (0.43-0.63)
		*Aβ*( 95%CI)		*Aβ( 95%CI)*		*Aβ( 95%CI)*		*Aβ( 95%CI)*		*Aβ( 95%CI)*		*Aβ( 95%CI)*	
HAZ^†^	0	0.20* (0.02-0.38)	0.58^***^ (0.42-0.75)	-0.11 (-0.33-0.11)	0.18 (-0.02-0.37)	0.35^***^ (0.17-0.53)	0.42^**^ (0.16-0.69)	0.21^*^ (0.04-0.39)	0.30^**^ (0.11-0.49)	0.12 (-0.08-0.32)	0.13 (-0.05-0.30)	0.21^***^ (0.13-0.29)	0.36^***^ (0.27-0.44)
WAZ^†^	0	0.28*** (0.15-0.42)	0.55^***^ (0.42-0.67)	0.26^**^ (0.09-0.43)	0.41^***^ (0.26-0.57)	0.19^**^ (0.05-0.32)	0.23^*^ (0.03-0.43)	0.30^***^ (0.16-0.43)	0.38^***^ (0.24-0.53)	0.19^*^ (0.03-0.34)	0.39^***^ (0.26-0.53)	0.26^***^ (0.20-0.32)	0.43^***^ (0.37-0.50)
WHZ^†^	0	0.24** (0.10-0.39)	0.28^***^ (0.14-0.41)	0.44^***^ (0.26-0.61)	0.42^***^ (0.26-0.57)	-0.07 (-0.21-0.07)	-0.10 (-0.30-0.10)	0.30^***^ (0.17-0.42)	0.34^***^ (0.20-0.47)	0.12 (-0.04-0.28)	0.43^***^ (0.30-0.57)	0.20^***^ (0.14-0.27)	0.31^***^ (0.24-0.37)

*AOR* adjusted odds ratio, *Aβ* adjusted linear regression coefficient, *CI* confidence interval, *HAZ* Z scores for height-for-age, *WAZ* Z scores for weight-for-age, *WHZ* Z scores for weight-height

^#^Two-level or three-level GLMMs with binominal link; ^†^ Two-level or three-level GLMMs with normal link

^Adjusted for child age and gender, father and mother’s educational attainment, household wealth index, caste/ethnicity, expected food shortage, family size, and clustering of children within wards. *p<0.05; ** *p*<0.01; ***p<0.001

### Immediate causes

Data on child diarrhoea, pneumonia, and care and health seeking behaviours are summarised in Table 5 and Additional file 1: Table S4. After controlling for socio-demographic and economic factors (Table 5), the prevalence of diarrhoea (AOR: 0.80, 95%CI: 0.66-0.98, p<0.05) among children in the Karnali zone declined overtime. It also declined in Dolpa, Jumla and Humla districts, but increased in Mugu district. Overall the prevalence of pneumonia (AOR: 2.79, 95%CI: 2.28-3.42, *p*<0.001) and fever (AOR 1.42, 95%CI: 1.17-1.72, *p*<0.001) among children increased significantly overtime in the Karnali zone. Analyses by districts found that the prevalence of pneumonia among children also increased across all districts except Mugu district while that of fever increased in Humla and Kalikot districts but remained stable in Dolpa, Jumla and Mugu districts.

**Table 5:** Trend in WASH, IYCF and child disease prevention and management in the Karnali Zone of Nepal after controlling for confounders^a^, 2009-2015

Variables	2009 (Ref.)	Dolpa AOR(95%CI)		Jumla AOR (95%CI)		Mugu AOR(95%CI)		Humla AOR(95%CI)		Kalikot AOR(95%CI)		Karnali Zone AOR(95%CI)	
		2013	2015	2013	2015	2013	2015	2013	2015	2013	2015	2013	2015
Water and sanitation													
‘Unimproved’ drinking water sources ^b^	1	0.05*** (0.03-0.07)	0.13*** (0.09-0.18)	0.27** (0.12-0.61)	1.08 (0.57-2.07)	1.26 (0.88-1.80)	0.60 (0.33-1.09)	1.99** (1.34-2.94)	0.24*** (0.15-0.40)	0.08*** (0.05-0.125)	0.22*** (0.16-0.30)	0.36*** (0.30-0.42)	0.29*** (0.24-0.34)
‘Inadequate’ water treatment method ^b^	1	2.05*** (1.38-3.04)	12.61*** (7.69-20.66)	1.15 (0.62-2.14)	0.91 (0.54-1.53)	2.53* (1.24-5.17)	0.46* (0.22-0.96)	0.89 (0.55-1.45)	0.94 (0.56-1.57)	1.24 (0.64 -2.40)	1.12 (0.64-1.95)	1.76*** (1.40-2.23)	1.85*** (1.47-2.33)
‘Unimproved’ sanitation facilities ^b^	1	0.37*** (0.26-0.53)	0.02*** (0.02-0.03)	0.01*** (0.01-0.02)	0.06*** (0.04-0.10)	0.01*** (0.003-0.01)	0.00*** (0.00-0.00)	0.06*** (0.04-0.09)	0.02*** (0.01-0.03)	0.02*** (0.01-0.03)	0.02*** (0.01-0.03)	0.04*** (0.04-0.05)	0.02*** (0.02-0.03)
‘Unsanitary’ disposal of children’s faeces ^b^	1	0.15*** (0.11-0.21)	0.12*** (0.09-0.16)	0.13*** (0.09-0.19)	0.15*** (0.11-0.21)	0.21*** (0.15-0.28)	0.14*** (0.09-0.21)	0.51*** (0.39-0.66)	0.18*** (0.13-0.24)	0.04*** (0.02-0.05)	0.06*** (0.04-0.08)	0.18*** (0.16-0.21)	0.13*** (0.11-0.15)
IYCF Practices													
Early initiation of breastfeeding ^b^	1	4.54*** (2.81-7.33)	1.67* (1.11-2.51)	1.12 (0.64-1.95)	1.94** (1.18-3.17)	0.57** (0.38-0.86)	1.00 (0.53-1.90)	0.46*** (0.31-0.69)	0.73 (0.45-1.16)	3.09*** (1.95-1.90)	1.71** (1.17-2.51)	1.17 (0.97-1.40)	1.28** (1.06-1.55)
Bottle feeding ^b^	1	1.47 (0.57-3.78)	8.25*** (3.75-18.16)	8.92** (1.75-45.05)	40.00*** (7.54-153.20)	13.86*** (5.24-36.68)	19.75*** (6.43-60.70)	8.64*** (2.67-28.00)	29.39*** (9.02-95.73)	5.49*** (2.33-12.94)	2.47* (1.07-5.69)	11.47*** (6.56-20.04)	24.72*** (14.22-42.97)
Prevalence													
Diarrhoea ^b^	1	0.83 (0.54-1.26)	0.35*** (0.23-0.52)	0.57 (0.32-1.02)	0.39*** (0.24-0.64)	4.27*** (2.43-7.51)	5.94*** (2.64-13.35)	0.82 (0.54-1.24)	0.60* (0.38-0.94)	2.60** (1.42-4.75)	1.50 (0.90-2.50)	1.11 (0.91-1.36)	0.80* (0.66-0.98)
Pneumonia ^b^	1	1.83** (1.18-2.82)	3.06*** (2.02-4.63)	3.11*** (1.69-5.73)	9.53*** (5.48-16.58)	1.26 (0.76-2.09)	0.52 (0.25-1.12)	1.52 (0.96-2.40)	4.03*** (2.48-6.54)	1.06 (0.59-1.90)	3.41*** (2.10-5.56)	1.79*** (1.46-2.21)	2.79*** (2.28-3.42)
Fever ^b^	1	0.81 (0.54-1.21)	1.20 (0.82-1.75)	0.96 (0.54-1.71)	1.10 (0.68-1.80)	1.23 (0.74-2.05)	1.38 (0.65-2.91)	1.19 (0.79-1.80)	1.84** (1.18-2.88)	1.29 (0.74-2.25)	2.70*** (1.67-4.27)	1.07 (0.88-1.30)	1.42*** (1.17-1.72)
Action taken													
Sought advice ^b^	1	2.44*** (1.79-3.32)	1.46* (1.09-1.94)	1.89** (1.30-2.73)	2.04*** (1.47-2.82)	1.98*** (1.37-2.87)	2.93)*** (1.77-4.85	2.01*** (1.51-2.67)	2.69*** (1.99-3.64)	2.60*** (1.77-3.81)	5.06*** (3.63-7.04)	1.93*** (1.68-2.23)	2.55*** (2.21-2.93)
More fluid ^b^	1	5.08*** (2.03-12.72)	1.66 (0.64-4.33)	1.15 (0.39-3.34)	3.17* (1.32-7.60)	3.43* (1.08-10.93)	2.00 (0.48-8.31)	1.22 (0.57-2.61)	1.23 (0.56-2.68)	1.91 (0.72-5.10)	2.03 (0.87-4.75)	2.37*** (1.57-3.58)	2.32*** (1.54-3.48)
More food^#^	1	6.32** (1.69-23.74)	3.63 (0.94-14.04)	1.99 (0.47-8.43)	3.29 (0.90-12.07)	13.10* (1.61-106.37)	12.95* (1.09-153.58)	0.46 (0.10-2.19)	1.06 (0.27-4.19)	1.00 (0.08-11.95)	4.34 (0.50-37.87)	2.59** (1.37-4.92)	2.55** (1.36-4.79)
More breastfeeding ^b^	1	4.26*** (2.33-7.80)	3.14*** (1.83-5.37)	77.03*** (a) (29.37-202.03)	21.21*** (10.16-44.26)	2.25* (1.14-4.47)	1.23 (0.48-3.14)	1.39 (0.81-2.39)	2.84*** (1.58-5.10)	9.77*** (4.22-22.63)	7.38*** (3.53-15.45)	4.19*** (3.22-5.45)	3.91 *** (3.03-5.05)
Prevention													
Deworming ^b^	1	4.20*** (2.24-7.87)	1.58 (0.85-2.65)	0.92 (0.37-2.32)	0.85 (0.37-1.93)	0.06*** (0.03-0.14)	0.29 (0.08-1.07)	2.14* (1.18-3.89)	6.35*** (2.99-13.49)	0.78 (0.39-1.53)	1.01 (0.54-1.90)	0.93 (0.69-1.24)	1.39* (1.03-1.88)
Iodised salt ^b^	1	1.34 (0.84-1.83)	5.42*** (3.46-8.49)	0.90 (0.53-1.55)	1.71 (0.95-3.09)	1.65** (1.24-2.19)	3.51*** (2.22-5.54)	0.77 (0.55-1.08)	3.56*** (2.09-6.08)	0.49*** (0.36-0.66)	0.68** (0.53-0.87)	0.90 (0.77-1.05)	1.67*** (1.42-1.96)

*AOR* adjusted odds ratio, *CI* confidence interval, *Ref* reference. ^b^ Two-level GLMMs with binominal link; ^#^ Two-level GLMMs with Poisson link

^a^Adjusted for child age and gender, father and mother’s educational attainment, household wealth index, caste/ethnicity, expected food shortage, family size, and clustering of children within wards

(a) The large AOR and its 95%CI are due to small sample sizes and the variance in data at each measurement time

*p<0.05; ** *p*<0.01; ***p<0.001

Overall, the proportion of mothers seeking medical advice (AOR: 2.55; 95%CI: 2.2, 2.93, p<0.001), and giving more fluid (AOR: 2.32; 95%CI: 1.54, 3.48, p<0.001), giving more food (AOR: 2.55, 95%CI: 1.36, 4.79) or breastfeeding more (AOR: 3.91, 95%CI: 3.03, 5.05) in response to child illness increased significantly over time in the Karnali zone (Table 5). An upward trend was also observed in the proportion of children dewormed (AOR: 1.39, 95%CI: 1.03, 1.88, *p*<0.05) and the proportion of households using iodised salt (AOR: 1.67, 95%CI: 1.42, 1.96, *p*<0.001). However, differences were observed between districts. The proportion of mothers giving more food in response to child illness increased significantly only in Mugu district, while no trend was found for giving more fluid across all district except Jumla district where an upward trend was observed. For child deworming, a positive trend was only depicted in Humla district. There was a positive trend for the use of iodised salt in all districts except Kalikot district where a downward trend was observed.

Data on food consumption and dietary diversity are summarised in Table 6 and Additional file 1: Table S5. No trend was depicted for the food consumption. There was a deterioration in dietary diversity, with the proportion of households reporting high dietary diversity decreased significantly by 5.7 percentage points, from 22.5% in 2009 to 19.2% in 2013 and 16.8% in 2015 (p<0.01). These findings remained consistent after adjusting for socio-demographic and economic factors across the Karnali zone and all districts except Humla and Kalikot districts (Table 6).

**Table 6:** Trend in household food consumption, dietary diversity, and food security in the Karnali Zone of Nepal after controlling for confounders, 2009-2015

Variables	2009 (Ref.)	Dolpa AOR(95%CI)		Jumla AOR(95%CI)		Mugu AOR(95%CI)		Humla AOR(95%CI)		Kalikot AOR(95%CI)		Karnali Zone AOR(95%CI)	
		2013	2015	2013	2015	2013	2013	2015	2015	2013	2015	2013	2015
Household food consumption ^†^													
Poor (ref)	1	1	1	1	1	1	1	1	1	1	1	1	1
Borderline	1	0.84 (0.57-1.24)	1.17 (0.82-1.68)	0.95 (0.58-1.54)	0.95 (0.62-1.46)	0.95 (0.54-1.67)	0.92 (0.63-1.35)	0.76 (0.53-1.08)	0.86 (0.59-1.26)	0.91 (0.60-1.39)	1.17 (0.81-1.68)	0.83 (0.60-1.16)	1.06 (0.76-1.47)
Acceptable	1	0.97 (0.68-1.39)	1.05 (0.76-1.45)	0.97 (0.63-1.48)	0.96 (0.66-1.40)	1.09 (0.79-1.52)	1.01 (0.62-1.65)	1.34 (0.97-1.84)	1.02 (0.72-1.45)	0.96 (0.66-1.39)	0.97 (0.70-1.35)	1.27 (0.92-1.74)	0.95 (0.69-1.30)
Household dietary diversity ^†^													
Low dietary diversity (ref)	1	1	1	1	1	1	1	1	1	1	1	1	1
Moderate dietary diversity	1	0.33^***^ (0.24-0.44)	0.37^***^ (0.28-0.49)	1.15 (0.78-1.69)	0.48^***^ (0.35-0.67)	1.39 (1.05-1.83)^*^	0.70 (0.46-1.06)	1.01 (0.78-1.32)	0.72^*^ (0.53-0.96)	0.96 (0.70-1.31)	0.79 (0.60-1.03)	0.73*** (0.64-0.83)	0.37*** (0.32-0.42)
High dietary diversity	1	0.24^***^ (0.17-0.34)	0.32)^***^ (0.24-0.44	0.62 (0.41-0.94)^*^	0.25^***^ (0.18-0.37)	1.28 (0.92-1.77)	0.59^*^ (0.37-0.95)	0.92 (0.67-1.25)	0.78 (0.56-1.10)	1.10 (0.76-1.60)	0.83 (0.60-1.16)	0.46^***^ (0.39-0.55)	0.21^***^ (0.18-0.25)
Household food security†													
Severely food insecure (ref)	1	1	1	1	1	1	1	1	1	1	1	1	1
Moderate food insecure	1	0.58 (0.37-0.90)	0.80 (0.51-1.26)	0.85 (0.48-1.50)	1.13 (0.68-1.87)	0.78 (0.50-1.23)	1.08 (0.50-2.30)	1.16 (0.82-1.64)	1.09 (0.72-1.63)	0.77 (0.45-1.29)	0.83 (0.53-1.31)	0.65*** (0.53-0.81)	0.94 (0.75-1.19)
Food secure	1	0.79 (0.52-1.20)	1.12 (0.73-1.72)	1.05 (0.63-1.72)	1.05 (0.67-1.64)	0.79 (0.52-1.20)	1.91 (0.97-3.76)	0.95 (0.70-1.30)	1.49^*^ (1.04-2.12)	0.94 (0.60-1.47)	1.11 (0.75-1.64)	0.69*** (0.58-0.82)	1.43*** (1.18-1.73)

*AOR* adjusted odds ratio, *CI* confidence interval, *Ref* reference

^†^Two-level GLMMs with multinominal link

^a^Adjusted for child age and gender, father and mother’s educational attainment, household wealth index, caste/ethnicity, expected food shortage, family size, and clustering of children within wards.

*p<0.05; ** *p*<0.01; ***p<0.001

### Underlying causes

Data on underlying causes of child malnutrition are summarised in Table 5 and Additional file 1: Table S4. Improvements were observed in WASH outcomes, notably access to clean water, improved sanitation facilities, and safe disposal of children faeces in the Karnali zone and across all districts. However, the proportion of household reporting inadequate water treatment methods increased over time overall, but improvement was observed in Mugu district which recorded a decline in the proportion of households reporting inadequate water treatment methods.

There were improvements in IYCF indicators. The proportion of mothers reporting ever breastfeeding their children and continued breastfeeding at one year remained very high. Unfortunately, no significant trend was depicted for ever breastfed, exclusive breastfeeding under 6 months, exclusive breastfeeding for infants 4–5 months, continued breastfeeding at 1 year, continued breastfeeding at 2 years, and the introduction of solid/semi-solid/soft foods at 6–8 months. After adjusting for socio-demographic and economic factors (Table 5), the proportion of early initiation of breastfeeding increased significantly over time in the Karnali zone and across all districts, except Mugu and Humla districts where the trend was not significant. However, the prevalence of bottle feeding also increased significantly over time across all districts and the Karnali zone

In terms of food security (Table 6 and Additional file 1: Table S5), the proportion of households classified as food secure increased steadily by 7 percentage points, increasing from 42.8% in 2009 to 46.8% in 2013 and 59.8% in 2015 (*p*<0.001). After adjusting for socio-demographic and economic factors, the trend remained significant in the Karnali zone overall (1.43, 95%CI: 1.18, 1.73, *p*<0.001), but became non-significant across all districts except Humla district where an upward trend remained significant (AOR: 1.49, 95%CI: 1.04, 2.12, *p*<0.05).

Data presented in Additional file 1: Table S6 suggest that the proportion of households producing their own food as staple food increased by 6.6 and 4.1 percentage points in Dolpa and Humla districts respectively, but decreased by approximately 10 percentage points in Kalikot district. It remained unchanged in Jumla and Mugu districts. After adjusting for socio-demographic and economic factors the trend for Dolpa and Kalikot districts remained consistently significant, but in Humla it became non-significant. Overall, however, the proportion of households purchasing foods increased significantly, while receipt of food aid decreased in the Karnali Zone and across all districts before and after adjusting for socio-demographic and economic factors. In relation to household resilience and productive capital, the proportion of family members out-migrating temporarily was relatively very low, but increased significantly over time by six to ninefold in Dolpa, Jumla, and Mugu districts, with an overall increase observed in the Karnali zone (AOR: 3.50, 95%CI: 1.96, 6.23, *p*<0.001; Table 7). The proportion of households borrowing money increased significantly in Mugu and Humla districts, but decreased in Kalikot district. No significant trend was observed for Dolpa and Jumla districts and the whole Karnali Zone. The proportion of households selling land was relatively small and remained constant during the study period. In contrast, the proportion of households selling agricultural assets increased significantly across all districts except Humla district where the trend was not significant. All districts experienced a significant decline in crop farming as a source of income (except Humla district). The decline of crop farming as a source of income was compensated by an increase in income from livestock farming.

**Table 7:** Trend in sources of staple foods, resilience indicators, reproductive capital indicators, and source of income the Karnali Zone of Nepal after controlling for confounders ^a^, 2009-2015

Variables	2009 (Ref.)	Dolpa AOR(95%CI)		Jumla AOR(95%CI)		Mugu AOR(95%CI)		Humla AOR(95%CI)		Kalikot AOR(95%CI)		Karnali Zone AOR(95%CI)	
		2013	2015	2013	2015	2013	2015	2013	2015	2013	2015	2013	2015
Source of staple food ^b^													
Own production	1	2.13** (1.22-3.70)	1.97** (1.20-3.24)	1.17 (0.60-2.26)	1.02 (0.59-1.78)	1.09 (0.63-1.89)	0.61 (0.26-1.46)	1.03 (0.68-1.54)	1.47 (0.90-2.38)	0.10 (0.05-0.17)***	0.17*** (0.09-0.30)	0.84 (0.68-1.04)	1.09 (0.87-1.36)
Purchasing	1	5.45*** (3.93-7.55)	92.66*** (a) (54.87-156.50)	4.07*** (2.83-5.84)	10.03*** (6.99-14.39)	1.42** (1.10-1.85)	1.55* (1.06-2.27)	0.98 (0.74-1.30)	8.78*** (6.47-11.92)	1.9*** (1.41-2.56)	2.50*** (1.93-3.25)	2.46*** (2.18-2.78)	6.06*** (5.30-6.93)
Food aid	1	0.09*** (0.06-0.13)	0.00(a) (0.00-1.50*10^192^)	0.06*** (0.04-0.12)	0.00(a) (0.00-6.98*10^230^)	1.53 (1.09-2.21)*	0.53* (0.28-0.98)	0.18*** (0.13-0.25)	0.00(a) (0.00-3.66*10^39^)	117.16*** (44.93-305.45)	1.03 (0.20-5.31)	0.33*** (0.29-0.39)	0.02*** (0.01-0.02)
Resilience indicators													
Outmigration^#^	1	4.01 (0.66-24.53)	6.02* (1.14-31.69)	5.26 (0.86-32.18)	8.50* (1.67-43.39)	3.16 (0.77-12.89)	9.03* (1.71-47.69)	2.08 (0.62-6.97)	2.42 (0.69-8.52)	0.88 (0.22-3.56)	0.90 (0.27-2.98)	2.08* (1.13-3.84)	3.50*** (1.96-6.23)
Borrowing money*	1	1.55** (1.17-2.06)	0.96 (0.74-1.25)	1.14 (0.82-1.57)	1.20 (0.90-1.60)	0.68** (0.51-0.89)	1.84** (1.23-2.75)	1.45** (1.10-1.91)	1.88*** (1.39-2.56)	0.32*** (0.24-0.44)	0.39*** (0.30-0.51)	0.82** (0.73-0.93)	0.97 (0.86-1.09)
Reproductive capital indicators^#^													
Selling land	1	3.41 (0.29-40.44)	0.84 (0.04-17.17)	5.25*10^9^(a) (0.00-nr)	1.37*10^10^(a) (0.00-nr)	0.35 (0.08-1.54)	0.19 (0.04-1.09)	1.97 (0.34-11.41)	0.76 (0.08-7.03)	1.44 (0.44-4.74)	0.31 (0.07-1.29)	1.39 (0.70-2.77)	0.85 (0.41-1.79)
Selling agricultural assets	1	5.49** (1.75-17.23)	4.53** (1.47-13.94)	5.25** (1.86-14.79)	4.31** (1.61-11.49)	2.31** (1.25-4.29)	1.43 (0.65-3.16)	2.19** (1.28-3.74)	1.32 (0.71-2.43)	0.63 (0.35-1.13)	0.43** (0.26-0.74)	1.50** (1.15-1.97)	1.31 (0.99-1.73)
Source of income													
Crop farming ^b^	1	3.27*** (2.46-4.34)	0.46*** (0.36-0.59)	0.65* (0.46-0.91)	0.36*** (0.26-0.49)	1.64*** (1.25-2.14)	0.40*** (0.27-0.58)	1.90*** (1.49-2.42)	1.25 (0.95-1.63)	2.34*** (1.73-3.16)	0.41*** (0.31-0.53)	1.79*** (1.59-2.01)	0.51*** (0.45-0.57)
Livestock farming^#^	1	0.26** (0.12-0.57)	1.90** (1.21-3.00)	3.85*** (1.97-7.54)	2.91** (1.53-5.56)	1.45 (0.81-2.62)	4.87*** (2.37-10.01)	1.16 (0.73-1.83)	1.77* (1.12-2.80)	3.17* (1.06-9.49)	13.92*** (5.62-34.46)	1.20 (0.92-1.57)	3.03*** (2.40-3.81)
Employment ^b^	1	0.41*** (0.27-0.62)	1.02 (0.73-1.43)	0.76 (0.51-1.13)	0.91 (0.65-1.29)	1.47* (1.05-2.06)	8.43*** (5.09-13.97)	0.54*** (0.42-0.71)	0.73* (0.55-0.98)	0.60** (0.43-0.85)	0.89 (0.67-1.19)	0.61*** (0.53-0.71)	1.08 (0.93-1.24)

*AOR* adjusted odds ratio, *CI* confidence interval, *Ref* reference

^b^Two-level GLMMs with binominal link; ^#^ Two-level GLMMs with Poisson link

^a^Adjusted for child age and gender, father and mother’s educational attainment, household wealth index, caste/ethnicity, expected food shortage, family size, and clustering of children within wards

nr: IBM SPSS did not report the upper limit of 95%CI

(a) The large AOR and its 95%CI are due to small sample sizes and the variance in data at each measurement time

*p<0.05; ** *p*<0.01; ***p<0.001

## Discussion

This is the first study to examine the impact of UCT program on individual- and household/community-level causes of child health in Nepal. The link between poverty and child malnutrition is well documented and is characterised by two distinctive features [26, 27]: 1) nutrition constitutes one of the main determinants of health and consequently many countries have set minimum health and nutrition standards to ensure good health for all their citizens; and 2) health and nutritional interventions enable human capital formation. Therefore, it is possible that cash transfer programs, supported by other government policies and targeted resource transfers aimed at poverty alleviation can have major nutritional impacts, but this assertion remains poorly tested in the literature. The unconditional child cash grant allowed us to address this gap

The study found that UCT, when embedded within a government sponsored cash transfer program for families and complemented by capacity building and behavioural change education improves child nutritional status. The evidence of cash transfer on child nutritional status comes predominantly from CCT. For example, recently, the Overseas Development Institute completed a systematic review examining the impact of cash transfers [3]. They identified 41 studies addressing child malnutrition, of which 27 (26 CCT and 1 UCT) were in Latin America, 11 (8 UCT and 3 CCT) in sub-Saharan Africa, two in South Asia (1 CCT and 1 UCT), and one in the Asia and pacific region (1 CCT). The study found that of the 13 studies reporting overall effects on child stunting only five CCT had a statistically significant effect on HAZ (from 0.07 to 0.41). The impact on wasting was limited. Of the five studies that reported the effect of cash transfer on wasting, only one CCT found a statistically significant reduction of wasting by 13 percentage points among children aged 12–24 months. The impact on underweight was also limited; with only one CCT out of eight studies (2 UCT and 6 CCT) reporting overall cash transfer effects on this indicator, with a reduction of the prevalence of underweight by 6.2 percentage points. These findings suggest that only CCT had an effect on child malnutrition and none of the UCT reported any impact. These findings mirror those reported in an earlier systematic review carried earlier by Leroy et al [4]. The authors reviewed the impact of CCT programs on child nutrition outcomes in Latin America: and found consistent evidence of a positive impact of CCT programs on child anthropometry. They found that the effects were generally larger for height compared to weight-related indicators; children’s exposure to the programs at a younger age, and in countries where the size of the transfer was larger.

However, there has been limited evidence on the impact of UCT on child nutrition. Our findings show that UCT improves child nutritional status, fills in a gap in the literature and contradicts the literature. Houngbe and colleagues^9^conducted a 2-arm cluster-randomized controlled trial incorporating 32 villages randomly assigned to either the intervention or the control group to examine the impact of a multiannual and seasonal UCT program on wasting stunting, and morbidity among children <36 months old in Tapoa Province, in the eastern region of Burkina Faso. The intervention targeted households classified as poor or very poor according to household economy approach criteria and provided seasonal monthly allowance of 10,000 West African Financial Community of Africa Francs (∼US$17) by mobile phone to mothers from July to November over 2 years (2013 and 2014). The authors found no effect of the multiannual, seasonal UCT program on wasting and stunting among children. Nonetheless, children in the intervention group had a lower risk of self-reported respiratory tract infections than did children in the control group.

We found that the UCT had a negative impact on dietary diversity and no impact on food consumption. Our findings are in conflict with the literature [3, 6]. A recent systematic review of 12 studies examining the impact of cash transfers on dietary intake, seven were found to have had at least one statistically significant improvement in dietary diversity [3]. We also found that the intervention reduced the risk of diarrheal diseases in children, which is consistent with the literature [11]. However, we found that the intervention was associated with an increase in the prevalence of pneumonia, contradicting recent studies showing that cash transfers have an impact respiratory tract infections in children [9].

We found that UCT improves WASH outcomes. While social protection programs have been widely recognised as a key instrument in tackling child malnutrition, its impact on WASH remains poorly documented. Our findings are consistent with the few available data. It has been estimated that lifting poverty by improving the economic situation of people living on < US$ 2.00 per day to living on ≥US$ 2.00 per day can prevent 51% of the risk of exposure to unimproved water and/or sanitation [28]. Existing evidence suggests that households receiving cash transfers have better access to sanitation facilities than those without benefits [29]; families receiving cash transfers experience fewer difficulties in paying for safe drinking water and have better hygiene behaviours than those in the control group [30, 31]. Our findings suggest that social protection programs may improve WASH outcomes by removing social and financial barriers and affecting behavioural changes, which is consistent with the literature [29, 32–34]

We found that the project had no impact on overall food security but had a positive impact of food availability characterised by an increase in food purchasing and income from livestock farming, and a decrease in receipt of food aid and crop farming. Our findings are partially supported by the literature. Emerging evidence suggests that cash transfers have an impact on several measures of food security including an increase in expenditures on food, increase in the number of meals per day, an increase in the consumption of nutrient-rich food items, and an increase in livestock [35, 36]. However, our findings of a decline in crop farming and lack of the project impact of the overall food security is not consistent with the literature [35, 36]. The main reasons being the use of different measures, differences in the conceptualisation of food security [35] and variance in regularity of the cash payments especially as a relatively generous, regular and predictable transfer is associated with increases in both the quantity and quality of food but a smaller, lumpy and irregular transfer has been found to have no effect [37].

Finally we found that UCT improved care and health seeking behaviours, which is consistent with the literature [7, 8]. Cash transfers have been found to increase the use of maternal and child health facilities [7, 8]. This could explain our finding related to the increase in mothers’ breastfeeding more and seeking medical advice when the child gets sick. These behaviours could have been enhanced by the capacity building and health promotion activities that were complemented by the child grant scheme. In terms of IYCF indicators, early initiation of breastfeeding improved and bottle feeding became more prevalent over time. Studies examining the impact of cash transfers on IYCF are scarce [32]. The most documented one is the Zambian Child Grant Program which reported a program impact of 18 percentage points, with children in treatment households more likely to have had the minimum required number of feedings [38]. However, data on the impact of cash transfers on age specific IYCF measures such as age-specific breastfeeding practices, continued breastfeeding at 1 year, bottle feeding or the introduction of solid, semi-solid or soft are lacking.

There are a number of limitations worth acknowledging. Large confidence intervals were observed for the following indicators: mothers’ breastfeeding more in response to child illness in Jumla district, households purchasing foods and receiving food aid as indicators of source of staple foods in Dolpa, Jumla, Humla and Kalikot districts; and selling land as an indicator of reproductive capital in Jumla district. The large confidence intervals could be due to the small sample size and imprecision in the prevalence of these indicators at each measurement time [39], suggesting that the samples did not provide a precise representation of the population mean. Findings related to these indicators need to be interpreted with caution. The child cash grant in Nepal was an unconditional cash transfer program. It has been suggested that cash transfers that are roughly less than 20% of household expenditure have mixed or no impact of child health outcomes [40]. Those with cash transfers approximating ≥20% the household expenditure report a significant improvement in child health outcomes. The child grant in Nepal was less than 20% of household expenditure, which together with the unconditionality of the cash transfer, could explain some of our negative or null findings. Measuring the impact of cash transfers on behaviours such as food consumption and food security is challenging given the time differences between the receipt of the transfer and the timing of the survey (favourable reporting of behaviours is possible when the survey is undertaken within a short time after receiving the transfer). The study is based on repeated cross-sectional surveys to depict a trend. In order to reduce threats to external validity, a number of measures were implemented. Cross-sectional socio-demographic and economic factors were controlled for in the regression models depicting trends. The child cash grant was embedded within existing universal social transfer programs hence ensuring continuity of participation and preventing the disruption in disbursements. However, the implementation of the project involved too many stakeholders with differing expectations and competing objectives, which might have hampered the effective implementation of the project.

## Conclusion

Notwithstanding these limitations, results point to the conclusion that unconditional child cash grants embedded within a government sponsored cash transfer program for families and complemented by capacity building for effective social protection improves child nutritional status, WASH outcomes, food availability and care and health seeking behaviours. Targeted resource transfers for families, augmented with a child sensitive social protection program and capacity building for social protection can address effectively child health and related behaviours. To improve and sustain the impact some considerations need to be made to increase the child cash payment amount to ≥ 20% of household expenditure and to institutionalise and roll out the capacity building program.

## Additional file

Additional file 1: **Table S1.** UNICEF-supported intervention to improve child health outcomes through CCG programs. **Table S2**: IYCF indicators. **Table S3A** Trend in HAZ, WAZ, and WHZ among children under five in the Karnali Zone of Nepal, 2009-2015. **Table S3B**. Trend in the prevalence of stunting, underweight, and wasting among children under five in the Karnali Zone of Nepal, 2009-2015. **Table S4**: Trend in WASH, IYCF and child disease prevention and management. **Table S5**. Trend in household food consumption, dietary diversity, and food security in the Karnali Zone of Nepal, 2009-2015. **Table S6**: Trend in sources of staple foods, resilience indicators, reproductive capital indicators, and source of income. (DOCX 75 kb)

## Abbreviations

CCG: Child cash grant
CCT: Conditional Cash Transfer
FCHVs: Female Community Health Volunteers
FCS: Food consumption scores
GLMMs: Generalized Linear Mixed Models
GoN: Government of Nepal’s
HAZ: Height-for-age Z-scores
HDDS: Household Dietary Diversity Scores
HFIAS: Household Food Insecurity Access Scale
HWI: Household Wealth Index
IYCF: Infant and Young Child Feeding
NRs: Nepalese rupees
TRTs: Targeted Resource Transfers
UCT: Unconditional Cash Transfer
VDC: Village Development Committee
WASH: Water, Hygiene and Sanitation
WAZ: Weight-for-age Z-scores
WHO: World Health Organization
WHZ: Weight-for-height Z-scores
