# Supplementary material for: The impact of unconditional child cash grant on child malnutrition and its immediate and underlying causes in five districts of the Karnali Zone, Nepal – A trend analysis
Source: Arch Public Health. 2019 May 29;77:24. doi: 10.1186/s13690-019-0352-2 (PMC6540561; doi:10.1186/s13690-019-0352-2)
Supplement: Supplementary file 1 — Table S1. UNICEF-supported intervention to improve child health outcomes through CCG programs. Table S2: IYCF indicators. Table S3A Trend in HAZ, WAZ, and WHZ among children under five in the Karnali Zone of Nepal, 2009-2015. Table S3B. Trend in the prevalence of stunting, underweight, and wasting among children under five in the Karnali Zone of Nepal, 2009-2015. Table S4: Trend in WASH, IYCF and child disease prevention and management. Table S5. Trend in household food consumption, dietary diversity, and food security in the Karnali Zone of Nepal, 2009-2015. Table S6: Trend in sources of staple foods, resilience indicators, reproductive capital indicators, and source of income. (DOCX 75 kb) [file 13690_2019_352_MOESM1_ESM.docx]

**Table S1.** UNICEF-supported intervention to improve child health outcomes through CCG programs.

| **Program Activities** | **No. of Activities per Year** | **Frequency of Delivery** | **Mode of Delivery** |
| --- | --- | --- | --- |
| Advocacy and Capacity Building |  |  |  |
| Program planning and review workshop | Planning undertaken Once every year, review workshop done for twice (during 2013 and 2015) | 3 times in each districts during the project period - Review twice (during mid-implementation and final review) | Planning Meeting Workshop |
| Training in Infant and Young Child Feeding (IYCF) to all health workers at district level | In 2011 (Starting year) and Refresher in 2015 | One IYCF Training of Trainer (ToT) and Cascading initially, review were taken annually and refresher training in 2015 | ToT jointly by Program Cooperation Agreement (PCA) partner as well as Ministry of Health (MoH), Refresher undertaken by MoH mechanisms |
| Training to all health workers at health facility (VDC) level | In 2011 (Starting year) and Refresher in 2015 | One IYCF TOT and Cascading initially, review were taken annually and refresher training in 2015 | ToT jointly by PCA partner as well as MoH, Refresher undertaken by MoH mechanisms |
| Training to Female Community Heath Volunteers (FCHV), Traditional Healers, teachers and community leaders | In 2011 (Starting year) and Refresher in 2015 | twice | training/orientation Implementation through FCHV (Government/MoH) mechanisms, facilitated by Non Government Organization (NGO) partners |
| Facilitate Mothers Group Meeting on Nutrition at community level (at least one in each ward) | Mothers Group meeting happens every month in each settlement through Female Community Heath Volunteers | 12 times/ year/FCHV in all districts | Through MoH mechanisms |
| Orientation to social mobilizers and community facilitators in all five districts of Karnali. | One a year (in 2012 and 2015) | twice | orientation through NGO partner |
| Conduct 1 day VDC level refresher training/program orientation to FCHV in each VDC in Karnali zone (Ensure IYCF-Cash Grant refresher conducted by Heath post/District Public Health Offices) | One training and One Refresher in 5 years | twice | Through MoH mechanisms |
| Awareness raising |  |  |  |
| Orientation/meeting to mother/caregivers in all 5 districts (134 VDCs) of Karnali and demonstration of preparation of complementary food lito (mixtures of roasted and grinded cereals, legumes) and jaulo (cooked rice and lentils with vegetables and ghee) for children | twice in 5 years | 2 | Implementation through FCHVs (Government/MoH) mechanisms, facilitated by PCA partners (NGO) |
| Dissemination of Behaviour Change Communication message during orientations at 5 districts (134 VDCs) of Karnali | 1 | 5 | Implementation through Government/MoH (Health facilities, Health workers, FCHVs, etc.) mechanisms, facilitated by PCA partners (NGO) |
| Production of radio program on nutrition and social protection promotion. | 1 | 5 | Through Local Frequency Modulation (FM) Radio stations facilitated by NGO partners |
| Broadcasting the Nutrition and Social Protection related Radio Programmes through FM Radios | 1 | 5 | Through Local FM stations facilitated by NGO partners |
| Conduct local food preparation and demo at mother group/school and Early Childhood Development (ECD) | 1 | 5 (Once every year) | Through Government/MoH (Health workers, FCHVs, etc.) mechanisms at community level |
| Partnership with community Radios for producing and broadcasting the weekly magazine on child feeding, caring, nutrition, hygiene and sanitation. | 1 | 365 days | Through Local FM stations facilitated by NGO partners |
| Daily Airing of Jingle Message from FM radios. | 1 | 365 days | Through Local FM stations facilitated by NGO partners |
| Promote nutritious food (eggs, Jaulo) for underweight children and iodized salt to all children 0–23 coming to Growth Monitoring and pregnant women coming to Ante-Natal Checkups | 1 time in five year | 1 | Implementation through Government/MoH (Health facilities, Health workers, FCHVs, etc.) mechanisms, facilitated by NGO partners |
| Mobilization of other community structures like women’s group |  |  |  |
| Conduct ward~~s~~ level discussion/meeting through community structure | 1 | 5 | Through Government/MoH mechanisms, facilitated by NGO partners |
| Conduct joint monitoring and Evaluation at VDC level from District Resource Group and NGO Partner | 1 | 2 | Through Government/MoH mechanisms, facilitated by NGO partners |
| Community Facilitator for mobilization of community groups/institutions (2 persons in each district for 5 months) | 1 | 3 times (2011, 2012, 2015) | Through Government/MoH mechanisms, facilitated by NGO partners |
| Community Level Dramas at cluster level in all 5 district of Karnali | 1 | 2 times in each clusters | Through Government/MoH mechanisms, facilitated by NGO partners |
| Distribution of Child Grant to all under 5 children (in every four months) by the Government of Nepal | 3 times per year | 3 times per year in all 5 districts every year | Through Local government (VDCs) |
| Birth Registration Campaign |  |  |  |
| Conduct VDC level birth registration campaign promoting birth registration within 35 days in all 134 VDCs of Karnali | 1 | 2 (in 2012 and 2015) in all 5 districts | Through Local Government at district and village levels (District Development Committee (and Village Development Committees), facilitated by NGO partners |
| Provide incentive for those families who do birth registration within 35 days of child birth | 1 | 1 times in all 5 districts | Through Local Government at district and village levels, facilitated by NGO partners |
| Community Facilitator for mobilization of community groups/institutions(2 persons in each district for 5 months) | 1 | 3 times (2011, 2012, 2015) | Through Government/MoH mechanisms, facilitated by NGO partners |
| System Strengthening to Management Information System (MIS) of Social Protection Schemes (Child Grant, Old age Pension, Single Women Allowances, etc.) |  |  |  |
| Provide technical support to DDC and KIRDARC for Information Technology support and MIS rollout in all five districts of Karnali through consultants. | 1 | 1 times in each districts |  |
| Community Facilitator for mobilization of community groups/institutions(2 persons in each district for 5 months) | 1 | 3 times (2011, 2012, 2015) | Through Government/MoH mechanisms, facilitated by NGO partners |
| Supervision and Monitoring including process Monitoring | Ongoing | ongoing | Through Government/MoH mechanisms |

**Table S2: IYCF indicators**

| **Indicator** | **Definition** |
| --- | --- |
| Ever breastfed | Proportion of children born in the last 24 months who were ever breastfed. |
| Early initiation of breastfeeding: | Proportion of children born in the last 24 months who were put to the breast within one hour of birth |
| Exclusive breastfeeding under 6 months: | Proportion of infants 0–5 months of age who are fed exclusively with breast milk |
| Exclusive breastfeeding for infants 4–5 months | Proportion of infants 4–5 months of age who are fed exclusively with breast milk. |
| Continued breastfeeding at 1 year: | Proportion of children 12–15 months of age who are fed breast milk |
| Continued breastfeeding at 2 years | Proportion of children 20–23 months of age who are fed breast milk |
| Bottle feeding | Proportion of children 0–23 months of age who are fed with a bottle. |
| Introduction of solid, semi-solid or soft foods: | Proportion of infants 6–8 months of age who receive solid, semi-solid or soft foods. |
| Minimum dietary diversity: | Proportion of children 6–23 months of age who receive foods from 4 or more food groups |
| Minimum meal frequency:* | Proportion of breastfed and non-breastfed children 6–23 months of age, who receive solid, semi-solid, or soft foods (but also including milk feeds for non-breastfed children) the minimum number of times or more |
| Minimum acceptable diet* | Proportion of children 6–23 months of age who receive a minimum acceptable diet (apart from breast milk) |
| Children ever breastfed*: | Proportion of children born in the last 24 months who were ever breastfed |
| Consumption of iron-rich or iron-fortified foods the previous day | Proportion of children 6–23 months of age who receive iron-rich foods or iron-fortified foods that is specially designed for infants and young children, or that is fortified in the home |
| Consumption of Vitamin A-rich foods the previous day | Proportion of children 6–23 months of age who receive Vitamin A-rich foods |
| Consumption of protein foods the previous day | Proportion of children 6–23 months of age who receive protein-rich foods |

**Table S3A Trend in HAZ, WAZ, and WHZ among children under five in the Karnali Zone of Nepal, 2009-2015**

| *District* | HAZ [mean(SD)] | | | | WAZ [mean(SD)] | | | | WHZ [mean(SD)] | | | |
| --- | --- | --- | --- | --- | --- | --- | --- | --- | --- | --- | --- | --- |
|  | **2009**  **N=3750** | **2013**  **N=3,750** | **2015**  **N=3,647** | *p* for time | **2009**  **N=3750** | **2013**  **N=3,750** | **2015**  **N=3,647** | *p* for time | **2009**  **N=3750** | **2013**  **N=3,750** | **2015**  **N=3,647** | *P* for time |
| Dolpa | -2.50(1.52) | -2.39(1.59) | -1.92(1.43) | <0.001 ^a^ | -1.78(1.17) | -1.45(1.12) | -1.20(1.07) | <0.001 ^a^ | -0.46(1.21) | -0.08(1.22) | -0.14(1.16) | <0.001 ^a^ |
| Jumla | -2.39(1.46) | -2.18(1.54) | -1.97(1.41) | <0.001 ^a^ | -2.08(1.14) | -1.66(1.16) | -1.52(1.09) | <0.001 ^a^ | -1.02(1.12) | -0.59(1.19) | -0.58(1.09) | <0.001 ^a^ |
| Mugu | -2.60(1.54) | -2.24(1.60) | -2.15(1.45) | <0.001 ^a^ | -1.91(1.20) | -1.72(1.20) | -1.54(1.10) | <0.001 ^a^ | -0.54(1.06) | -0.59(1.29) | -0.45(1.17) | 0.060^a^ |
| Humla | -2.35(1.67) | -2.14(1.46) | -2.08(1.40) | 0.001^a^ | -2.02(1.26) | -1.74(1.16) | -1.63(1.09) | <0.001 ^a^ | -0.97(1.12) | -0.70(1.10) | -0.62(1.11) | <0.001 ^a^ |
| Kalikot | -2.55(1.41) | -2.16(1.61) | -2.23(1.38) | <0.001 ^a^ | -2.06(1.12) | -1.80(1.11) | -1.59(1.10) | <0.001 ^a^ | -0.82(1.14) | -0.78(1.30) | -0.43(1.01) | <0.001 ^a^ |
| Karnali Zone | -2.48(1.52) | -2.22(1.56) | -2.07(1.42) | <0.001^b^ | -1.97(1.18) | -1.67(1.16) | -1.47(1.10) | <0.001^b^ | -0.76(1.15) | -0.55(1.24) | -0.42(1.13) | <0.001^b^ |

HAZ= Z scores for height-for-age; WAZ= Z scores for weight-for-age; WHZ= Z scores for weight-height; SD=standard deviation.

^a^ Two-level GLMMs with normal link were used to adjust for clustering of children within wards. ^b^ Three-level GLMMs with normal link were used to adjust for clustering within districts and clustering of children within wards.

**Table S3B. Trend in the prevalence of stunting, underweight, and wasting among children under five in the Karnali Zone of Nepal, 2009-2015**

| District | Stunting (%) | | | | Underweight (%) | | | | Wasting (%) | | | |
| --- | --- | --- | --- | --- | --- | --- | --- | --- | --- | --- | --- | --- |
|  | 2009  N=3750 | 2013  N=3,750 | 2015  N=3,647 | p for time | 2009  N=3750 | 2013  N=3,750 | 2015  N=3,647 | p for time | 2009  N=3750 | 2013  N=3,750 | 2015  N=3,647 | P for time |
| Dolpa | 65.1 | 60.8 | 54.8 | <0.001a | 42.6 | 36.2 | 28.7 | <0.001 a | 9.0 | 10.3 | 5.7 | 0.094 a |
| Jumla | 63.6 | 58.3 | 53.0 | 0.002 a | 54.5 | 38.3 | 36.7 | <0.001 a | 16.3 | 11.1 | 8.4 | <0.001 a |
| Mugu | 68.6 | 58.4 | 59.0 | <0.001 a | 46.9 | 40.2 | 33.3 | <0.001 a | 8.0 | 12.0 | 9.4 | 0.066a |
| Humla | 64.0 | 54.8 | 57.9 | 0.001 a | 52.9 | 41.3 | 38.4 | <0.001 a | 16.8 | 9.8 | 9.8 | <0.001 a |
| Kalikot | 66.7 | 57.9 | 59.8 | 0.002 a | 50.7 | 41.6 | 34.8 | <0.001 a | 12.7 | 14.3 | 5.7 | <0.001 a |
| Karnali Zone | 65.7 | 58.6 | 56.3 | <0.001b | 49.0 | 38.4 | 32.5 | <0.001b | 12.5 | 10.7 | 7.4 | <0.001b |

^a^ Two-level GLMMs with binomial link were used to adjust for clustering of children within wards. ^b^ Three-level GLMMs with binomial link were used to adjust for clustering within districts and clustering of children within wards.

**Table S4: Trend in WASH, IYCF and child disease prevention and management**

| Variables | Dolpa | | | | Jumla | | | | Mugu | | | |
| --- | --- | --- | --- | --- | --- | --- | --- | --- | --- | --- | --- | --- |
|  | 2009  N=750 | 2013  N=750 | 2015  N=947 | p for time a | 2009  N=750 | 2013  N=750 | 2015  N=575 | p for time a | 2009  N=750 | 2013  N=750 | 2015  N=750 | P for time a |
| Water and sanitation *(%/n)* |  |  |  |  |  |  |  |  |  |  |  |  |
| ‘Unimproved’ sources of drinking water* | 39.9/750 | 8.4/750 | 11.6/947 | <0.001 | 3.5/750 | 8.4/750 | 8.0/575 | <0.001 | 16.8/750 | 14.9/750 | 8.1/750 | <0.001 |
| ‘Inadequate’ water treatment method* | 80.7/750 | 76.9/750 | 96.0/947 | <0.001 | 93.1/750 | 89.6/750 | 87.7/575 | 0.036 | 95.9/750 | 97.1/750 | 83.3/750 | <0.001 |
| ‘Unimproved’ sanitation facilities* | 88.9/750 | 63.2/750 | 22.2/947 | <0.001 | 92.7/750 | 16.8/750 | 47.0/575 | <0.001 | 98.0/750 | 41.6/750 | 16.9/750 | <0.001 |
| ‘Unsanitary’ disposal of children’s faeces* | 75.9/750 | 33.9/750 | 30.1/947 | <0.001 | 67.6/750 | 22.4/750 | 25.7/575 | <0.001 | 81.1/750 | 46.5/750 | 23.5/750 | <0.001 |
| IYCF Practices *(%/n)* |  |  |  |  |  |  |  |  |  |  |  |  |
| Ever breastfed^#^ | 100.0/264^ | 100.0/257 | 100.0/361 | - | 100.0/284 | 99.7/343 | 99.6/274 | 0.999 | 99.3/285 | 100.0/326 | 99.7/324 | 0.996 |
| Early initiation of breastfeeding* | 46.6/264 | 76.7/257 | 57.8/360 | <0.001 | 68.3/284 | 77.9/340 | 82.4/273 | <0.001 | 60.2/279 | 51.2/326 | 75.2/323 | <0.001 |
| Exclusive breastfeeding under 6 months* | 55.6/54 | 65.3/49 | 76.7/60 | 0.066 | 80.3/61 | 79.1/67 | 87.3/55 | 0.475 | 75.4/57 | 79.2/72 | 73.1/52 | 0.730 |
| Exclusive breastfeeding for infants 4–5 months* | 33.3/21 | 45.5/22 | 66.7/27 | 0.079 | 59.3/27 | 68.0/25 | 83.3/18 | 0.258 | 47.8/23 | 59.3/27 | 58.3/24 | 0.749 |
| Continued breastfeeding at 1 year^#^ | 94.3/53 | 100.0/52 | 94.5/73 | 0.943 | 98.4/63 | 97.1/68 | 95.4/64 | 0.996 | 100.0/47 | 97.1/70 | 98.2/57 | 0.988 |
| Continued breastfeeding at 2 years* | 97.4/39 | 91.7/36 | 82.0/61 | 0.125 | 97.1/34 | 86.7/60 | 84.8/46 | 0.350 | 89.1/46 | 87.0/46 | 87.0/54 | 0.942 |
| Bottle feeding* | 1.1/264 | 4.3/257 | 23.6/360 | <0.001 | 0.7/284 | 7.3/343 | 21.2/274 | <0.001 | 2.1/285 | 20.3/325 | 31.8/324 | <0.001 |
| Intro of solid/semi-solid/soft foods at 6–8 months* | 96.9/32 | 83.3/36 | 89.8/49 | 0.315 | 93.3/30 | 87.5/32 | 100.0/24 | 0.460 | 92.9/28 | 90.3/31 | 93.2/44 | 0.923 |
| Intro of solid/semi-solid/soft foods at <6 months* | 96.4/28 | 32.7/49 | 20.0/60 | <0.001 | 83.3/12 | 19.4/67 | 10.7/56 | <0.001 | 100.0/9 | 20.8/72 | 21.2/52 | 0.984 |
| Prevalence *(%/n)* |  |  |  |  |  |  |  |  |  |  |  |  |
| Diarrhoea* | 53.5/286 | 53.3/304 | 35.0/371 | <0.001 | 57.0/207 | 42.1/254 | 32.6/215 | <0.001 | 22.0/150 | 51.0/202 | 57.7/267 | <0.001 |
| Pneumonia* | 36.0/286 | 43.1/304 | 49.9/371 | 0.002 | 25.6/207 | 56.3/254 | 77.2/215 | <0.001 | 40.0/150 | 50.0/202 | 34.1/267 | 0.003 |
| Fever* | 43.7/286 | 38.8/304 | 46.6/371 | 0.064 | 48.3/207 | 43.3/254 | 50.2/215 | 0.298 | 51.3150 | 50.5/202 | 39.0/267 | 0.011 |
| Action taken (*%/n)* |  |  |  |  |  |  |  |  |  |  |  |  |
| Sought advice* | 17.6/750 | 29.5/750 | 23.2/947 | <0.001 | 17.1/750 | 27.2/750 | 30.6/575 | <0.001 | 8.1/750 | 16.9/750 | 25.1/750 | <0.001 |
| More fluid* | 3.4/262 | 15.5/304 | 5.1/371 | <0.001 | 3.8/186 | 11.4/254 | 21.9/215 | <0.001 | 3.2/124 | 16.3/202 | 16.5/267 | 0.007 |
| More food^#^ | 1.5/263 | 9.2/304 | 4.0/371 | 0.001 | 1.9/186 | 9.1/254 | 10.7/215 | 0.017 | 0.8/119 | 7.4/202 | 6.0/267 | 0.106 |
| More breastfeeding* | 21.2/260 | 54.8/126 | 46.9/209 | <0.001 | 15.1/199 | 73.3/172 | 61.0/154 | <0.001 | 21.6/102 | 46.9/130 | 45.9/229 | <0.001 |
| Prevention *(%/n)* |  |  |  |  |  |  |  |  |  |  |  |  |
| Received Vitamin A capsules^#^ | 87.8/662 | 90.8/698 | 89.2/886 | 0.835 | 99.4/656 | 99.3/683 | 98.6/518 | 0.991 | 98.8/686 | 90.1/677 | 98.3/697 | 0.183 |
| Deworming* | 86.8/593 | 89.9/625 | 87.4/777 | 0.466 | 99.5/586 | 98.3/593 | 97.8/455 | 0.693 | 98.0/610 | 89.0/600 | 97.8/604 | <0.001 |
| Iodised salt* | 82.5/750 | 79.2/750 | 92.4/947 | <0.001 | 85.5/750 | 92.9/750 | 96.2/575 | <0.001 | 56.4/750 | 73.7/750 | 88.0/750 | <0.001 |
| Updated Immunisation status^#^ | 88.4/750 | 95.5/750 | 95.0/947 | 0.271 | 99.2/750 | 99.3/750 | 99.0/575 | 0.998 | 95.3/750 | 95.6/750 | 97.6/750 | 0.887 |
| DPT immunisation^#^ | 96.6/656 | 97.6/716 | 96.6/900 | 0.973 | 98.7/744 | 98.0/745 | 96.8/569 | 0.947 | 97.7/708 | 94.1/715 | 99.0/732 | 0.618 |
| Measles immunisation^#^ | 87.9/580 | 97.0/643 | 96.4/806 | 0.184 | 97.5/650 | 97.2/651 | 98.2/495 | 0.987 | 94.7/619 | 93.6/641 | 94.5/650 | 0.979 |

**Table S4: Continued**

| Variables | Humla | | | | Kalikot | | | | Karnali Zone | | | |
| --- | --- | --- | --- | --- | --- | --- | --- | --- | --- | --- | --- | --- |
|  | **2009**  **N=750** | **2013**  **N=750** | **2015**  **N=625** | *p* for time ^a^ | **2009**  **N=750** | **2013**  **N=750** | **2015**  **N=750** | *p* for time ^a^ | **2009**  **N=3750** | **2013**  **N=3,750** | **2015**  **N=3,647** | *P* for time ^b^ |
| Water and sanitation *(%/n)* |  |  |  |  |  |  |  |  |  |  |  |  |
| ‘Unimproved’ sources of drinking water* | 10.7/750 | 24.7/750 | 8.3/625 | <0.001 | 32.0/750 | 13.5/750 | 15.7/750 | <0.001 | 20.6/3750 | 14.0/3750 | 10.6/3647 | <0.001 |
| ‘Inadequate’ water treatment method* | 96.7/750 | 94.0/750 | 93.3/625 | 0.124 | 96.4/750 | 97.2/750 | 96.9/750 | 0.885 | 92.5/3750 | 91.0/3750 | 91.8/3647 | 0.073 |
| ‘Unimproved’ sanitation facilities* | 91.7/750 | 50.0/750 | 21.9/625 | <0.001 | 94.1/750 | 18.1/750 | 21.7/750 | <0.001 | 93.1/3750 | 37.9/3750 | 24.9/3647 | <0.001 |
| ‘Unsanitary’ disposal of children’s faeces* | 67.9/750 | 53.7/750 | 23.7/625 | <0.001 | 82.3/750 | 15.5/750 | 25.5/750 | <0.001 | 74.9/3750 | 34.4/3750 | 26.0/3647 | <0.001 |
| IYCF Practices *(%/n)* |  |  |  |  |  |  |  |  |  |  |  |  |
| Ever breastfed^#^ | 100.0/301 | 100.0/270 | 99.5/207 | 0.998 | 99.0/315 | 99.7/315 | 100.0/302 | 0.993 | 99.7/1449 | 99.9/1511 | 99.8/1468 | 0.998 |
| Early initiation of breastfeeding* | 72.4/301 | 51.1/270 | 62.6/206 | <0.001 | 48.5/291 | 71.8/312 | 60.5/301 | <0.001 | 59.5/1419 | 65.8/1505 | 67.5/1463 | <0.001 |
| Exclusive breastfeeding under 6 months* | 63.5/52 | 63.3/60 | 60.6/33 | 0.942 | 78.0/50 | 89.2/65 | 94.4/54 | 0.065 | 70.8/274 | 76.0/313 | 79.9/254 | 0.066 |
| Exclusive breastfeeding for infants 4–5 months* | 37.5/24 | 36.8/19 | 33.3/15 | 0.986 | 72.7/33 | 80.0/25 | 89.3/28 | 0.298 | 52.3/128 | 59.3/118 | 68.8/112 | 0.019 |
| Continued breastfeeding at 1 year^#^ | 100.0/56 | 100.0/52 | 98.1/54 | 0.994 | 97.9/48 | 97.0/67 | 94.9/59 | 0.986 | 98.1/267 | 98.1/309 | 96.7/307 | 0.984 |
| Continued breastfeeding at 2 years* | 94.7/38 | 89.4/47 | 85.0/40 | 0.474 | 97.0/66 | 85.2/54 | 89.3/56 | 0.151 | 95.1/223 | 87.7/243 | 85.6/257 | 0.015 |
| Bottle feeding* | 1.3/301 | 8.9/270 | 25.1/207 | <0.001 | 0.6/315 | 13.4/314 | 6.3/302 | <0.001 | 1.2/1449 | 11.1/1509 | 21.6/1467 | <0.001 |
| Intro of solid/semi-solid/soft foods at 6–8 months* | 88.6/35 | 100.0/30 | 100.0/30 | 0.290 | 87.5/24 | 82.4/34 | 85.4/48 | 0.860 | 91.9/149 | 88.3/163 | 92.3/195 | 0.422 |
| Intro of solid/semi-solid/soft foods at <6 months* | 82.4/17 | 36.7/60 | 39.4/33 | 0.014 | 66.7/9 | 10.8/65 | 5.6/54 | 0.001 | 88.0/75 | 23.3/313 | 17.6/255 | <0.001 |
| Prevalence *(%/n)* |  |  |  |  |  |  |  |  |  |  |  |  |
| Diarrhoea* | 48.9/113 | 45.2/281 | 36.3/256 | 0.027 | 25.7/148 | 40.6/219 | 35.5/324 | 0.017 | 44.5/1022 | 46.7/1260 | 39.2/1433 | 0.001 |
| Pneumonia* | 25.5/231 | 34.5/281 | 53.5/256 | <0.001 | 32.4/148 | 41.6/219 | 63.0/324 | <0.001 | 31.6/1022 | 44.7/1260 | 54.6/1433 | <0.001 |
| Fever* | 42.4/113 | 42.7/281 | 51.6/256 | 0.148 | 41.2/148 | 44.3/219 | 62.7/324 | <0.001 | 45.1/1022 | 43.4/1260 | 50.2/1433 | 0.002 |
| Action taken (*%/n)* |  |  |  |  |  |  |  |  |  |  |  |  |
| Sought advice* | 15.6/750 | 27.7/750 | 36.5/625 | <0.001 | 10.1/750 | 19.1/750 | 33.1/750 | <0.001 | 13.7/3750 | 24.1/3750 | 29.1/3647 | <0.001 |
| More fluid* | 5.6/196 | 9.6/281 | 12.1/256 | 0.136 | 6.5/124 | 8.7/219 | 14.2/324 | 0.029 | 4.4/892 | 12.3/1260 | 13.0/1433 | <0.001 |
| More food^#^ | 3.0/165 | 1.1/281 | 3.5/256 | 0.195 | 0.9/110 | 1.4/219 | 5.9/324 | 0.006 | 1.7/819 | 5.7/1260 | 5.7/1433 | <0.001 |
| More breastfeeding* | 27.3/209 | 33.5/161 | 47.4/152 | 0.001 | 10.4/115 | 48.1/156 | 44.9/236 | <0.001 | 19.9/885 | 51.7/745 | 48.5/980 | <0.001 |
| Prevention *(%/n)* |  |  |  |  |  |  |  |  |  |  |  |  |
| Received Vitamin A capsules^#^ | 92.9/662 | 95.5/690 | 99.3/591 | 0.509 | 97.4/683 | 94.7/685 | 95.6/676 | 0.880 | 95.3/3349 | 94.1/3433 | 95.6/3368 | 0.688 |
| Deworming* | 92.3/582 | 94.7627 | 97.2/541 | <0.001 | 97.2/598 | 94.2/602 | 96.5/600 | 0.228 | 94.8/2969 | 93.2/3047 | 94.7/2977 | 0.001 |
| Iodised salt* | 85.1/750 | 83.9/750 | 99.2/625 | <0.001 | 47.9/750 | 43.5/750 | 47.2/750 | 0.109 | 71.5/3750 | 74.6/3750 | 84.0/3647 | <0.001 |
| Updated Immunisation status^#^ | 98.1/750 | 96.5/750 | 99.5/625 | 0.855 | 96.5/750 | 97.9/750 | 98.4/750 | 0.931 | 95.5/3750 | 97.0/3750 | 97.6/3647 | 0.551 |
| DPT immunisation^#^ | 98.4/735 | 97.0/722 | 98.4/622 | 0.952 | 98.9/713 | 94.4/729 | 98.8/738 | 0.605 | 98.1/3556 | 96.2/3627 | 97.9/3561 | 0.671 |
| Measles immunisation^#^ | 95.3/621 | 94.0/647 | 97.0/561 | 0.868 | 94.7/625 | 96.4/642 | 96.9/643 | 0.918 | 94.2/3095 | 95.7/3224 | 96.5/3155 | 0.585 |

^a^ Two-level GLMMs with ^*^binominal, or ^#^ Poisson link were used to adjust for clustering of children within wards. ^b^ Three-level GLMMs with ^*^ binominal, or ^#^ Poisson link were used to adjust for clustering within districts and clustering of children within wards. .

**Table S5. Trend in household food consumption, dietary diversity, and food security in the Karnali Zone of Nepal, 2009-2015**

| Household food consumption | | | | | | | | | | |
| --- | --- | --- | --- | --- | --- | --- | --- | --- | --- | --- |
| District | **Poor (%)** | | | **Borderline(%)** | | | **Acceptable(%)** | | |  |
|  | **2009/2010** | **2013** | **2015** | **2009/2010** | **2013** | **2015** | **2009/2010** | **2013** | **2015** | ***p* for time** |
| Dolpa^a^ | 3.3 | 3.2 | 1.3 | 24.3 | 14.8 | 25.4 | 72.4 | 82.0 | 73.3 | 0.084 |
| Jumla^a^ | 2.1 | 1.6 | 2.4 | 19.3 | 15.6 | 17.0 | 78.5 | 82.8 | 80.5 | 0.935 |
| Mugu^a^ | 3.6 | 1.2 | 2.1 | 17.6 | 12.8 | 12.7 | 78.8 | 86.0 | 85.2 | 0.718 |
| Humla^a^ | 6.1 | 1.7 | 6.1 | 28.8 | 13.3 | 23.2 | 65.1 | 84.9 | 70.7 | 0.003 |
| Kalikot^a^ | 4.5 | 6.5 | 4.7 | 19.3 | 21.1 | 28.9 | 76.1 | 72.4 | 66.4 | 0.175 |
| Karnali Zone^b^ | 3.9 | 2.9 | 3.2 | 21.9 | 15.5 | 21.8 | 74.2 | 81.6 | 75.0 | <0.001 |
| Household dietary diversity | | | | | | | | | | |
| District | **Low dietary diversity(%)** | | | **Moderate dietary diversity(%)** | | | **High dietary diversity(%)** | | |  |
|  | **2009/2010** | **2013** | **2015** | **2009/2010** | **2013** | **2015** | **2009/2010** | **2013** | **2015** | ***p* for time** |
| Dolpa^a^ | 18.1 | 52.1 | 54.1 | 51.5 | 38.3 | 32.9 | 30.4 | 9.6 | 13.0 | <0.001 |
| Jumla^a^ | 17.7 | 14.8 | 47.5 | 43.9 | 52.8 | 33.7 | 38.4 | 32.4 | 18.8 | <0.001 |
| Mugu^a^ | 42.5 | 28.1 | 39.2 | 42.9 | 52.0 | 38.5 | 14.5 | 19.9 | 22.3 | <0.001 |
| Humla^a^ | 37.1 | 36.9 | 39.7 | 46.1 | 48.5 | 40.6 | 16.8 | 14.5 | 19.7 | 0.472 |
| Kalikot^a^ | 42.4 | 39.2 | 50.9 | 45.2 | 41.3 | 37.1 | 12.4 | 19.5 | 12.0 | 0.028 |
| Karnali Zone^b^ | 31.6 | 34.2 | 46.9 | 45.9 | 46.6 | 36.4 | 22.5 | 19.2 | 16.8 | 0.005 |
| Household food insecurity | | | | | | | | | | |
| District | **Severely food insecure(%)** | | | **Moderate food insecure(%)** | | | **Food secure(%)** | | |  |
|  | **2009/2010** | **2013** | **2015** | **2009/2010** | **2013** | **2015** | **2009/2010** | **2013** | **2015** | ***p* for time** |
| Dolpa^a^ | 34.0 | 38.2 | 29.5 | 31.1 | 16.5 | 19.9 | 34.9 | 45.3 | 50.5 | 0.022 |
| Jumla^a^ | 29.8 | 20.7 | 17.6 | 21.7 | 15.2 | 25.6 | 48.5 | 64.1 | 56.8 | 0.050 |
| Mugu^a^ | 36.8 | 39.5 | 11.9 | 25.3 | 22.6 | 14.9 | 37.8 | 37.9 | 73.2 | <0.001 |
| Humla^a^ | 44.1 | 38.8 | 24.5 | 15.9 | 22.9 | 16.0 | 40.0 | 38.4 | 59.5 | <0.001 |
| Kalikot^a^ | 27.0 | 29.9 | 25.0 | 20.6 | 17.5 | 16.4 | 52.3 | 52.6 | 58.6 | 0.791 |
| Karnali Zone^b^ | 35.0 | 33.9 | 22.0 | 22.2 | 19.2 | 18.2 | 42.8 | 46.8 | 59.8 | <0.001 |

^a^ Two-level GLMMs with multinominal link were used to adjust for clustering of children within wards. ^b^ Three-level GLMMs with multinominal link were used to adjust for clustering within districts and clustering of children within wards.

**Table S6: Trend in sources of staple foods, resilience indicators, reproductive capital indicators, and source of income.**

| Sources of staple foods | | | | | | | | | | | | |
| --- | --- | --- | --- | --- | --- | --- | --- | --- | --- | --- | --- | --- |
| District | **Own production (%)** | | | | **Purchasing(%)** | | | | **Food aid(%)** | | | |
|  | **2009/10** | **2013** | **2015** | **p for time** | **2009/10** | **2013** | **2015** | **p for time** | **2009/10** | **2013** | **2015** | **P for time** |
| Dolpa^a^ | 87.6 | 93.7 | 94.2 | <0.001 | 39.1 | 81.5 | 97.9 | <0.001 | 68.1 | 14.9 | 0.0 | <0.001 |
| Jumla^a^ | 91.1 | 92.0 | 90.1 | 0.302 | 44.7 | 79.6 | 88.3 | <0.001 | 42.0 | 5.1 | 0.0 | <0.001 |
| Mugu^a^ | 93.6 | 93.7 | 94.3 | 0.667 | 44.3 | 50.9 | 57.6 | 0.001 | 14.4 | 16.1 | 4.8 | <0.001 |
| Humla^a^ | 87.7 | 87.7 | 91.8 | 0.004 | 23.6 | 28.3 | 75.4 | <0.001 | 59.7 | 31.9 | 0.0 | <0.001 |
| Kalikot^a^ | 96.7 | 79.7 | 86.8 | <0.001 | 42.8 | 68.4 | 72.5 | <0.001 | 0.8 | 17.1 | 0.3 | <0.001 |
| Karnali Zone^b^ | 91.3 | 89.4 | 91.6 | 0.002 | 38.9 | 61.7 | 79.0 | <0.001 | 37.0 | 17.0 | 1.0 | <0.001 |
| Resilience indicators | | |  |  |  |  |  |  |  |  |  |  |
| District | **Outmigration(%)** | | | | **Borrowing money(%)** | | | | **Food shortage(%)** | | | |
|  | **2009/10** | **2013** | **2015** | **p for time** | **2009/10** | **2013** | **2015** | **p for time** | **2009/10** | **2013** | **2015** | **P for time** |
| Dolpa^a^ | 0.3 | 0.8 | 1.3 | 0.111 | 32.1 | 45.1 | 29.7 | <0.001 | 56.5 | 59.5 | 44.2 | <0.001 |
| Jumla^a^ | 0.4 | 1.2 | 2.1 | 0.033 | 46.7 | 44.5 | 49.2 | 0.113 | 59.3 | 75.3 | 66.4 | <0.001 |
| Mugu^a^ | 0.5 | 1.1 | 2.8 | 0.002 | 37.3 | 34.5 | 51.3 | <0.001 | 54.1 | 66.1 | 63.9 | <0.001 |
| Humla^a^ | 0.7 | 1.5 | 2.4 | 0.042 | 59.2 | 67.7 | 62.4 | 0.029 | 81.5 | 90.7 | 85.6 | <0.001 |
| Kalikot^a^ | 0.8 | 1.3 | 1.3 | 0.483 | 54.8 | 39.6 | 46.5 | <0.001 | 64.0 | 74.5 | 84.4 | <0.001 |
| Karnali Zone^b^ | 0.5 | 1.2 | 1.9 | <0.001 | 46.0 | 46.3 | 46.3 | 0.401 | 63.1 | 73.2 | 67.1 | <0.001 |
| Reproductive capital indicators | | | | | | | | | | | | |
| District | **Selling land(%)** | | | | **Selling household assets(%)** | | | | **Selling agricultural assets(%)** | | | |
|  | **2009/10** | **2013** | **2015** | **p for time** | **2009/10** | **2013** | **2015** | **p for time** | **2009/10** | **2013** | **2015** | **P for time** |
| Dolpa^a^ | 0.1 | 0.4 | 0.1 | 0.407 | 0.3 | 0.1 | 0.7 | 0.165 | 0.5 | 3.5 | 2.9 | 0.002 |
| Jumla^a^ | 0.1 | 1.1 | 0.5 | 0.113 | 0.0 | 0.4 | 0.5 | 0.745 | 0.8 | 3.5 | 4.0 | 0.002 |
| Mugu^a^ | 0.5 | 0.7 | 1.6 | 0.082 | 0.0 | 1.6 | 1.5 | 0.835 | 2.0 | 7.6 | 7.2 | <0.001 |
| Humla^a^ | 0.4 | 0.7 | 0.6 | 0.761 | 0.8 | 1.5 | 0.3 | 0.106 | 3.1 | 7.2 | 4.8 | 0.002 |
| Kalikot^a^ | 0.9 | 2.0 | 0.5 | 0.041 | 0.5 | 1.2 | 0.5 | 0.321 | 7.9 | 4.0 | 3.3 | 0.001 |
| Karnali Zone^b^ | 0.4 | 1.0 | 0.7 | 0.024 | 0.3 | 1.0 | 0.7 | 0.001 | 2.9 | 5.1 | 4.4 | <0.001 |
| Source of income. | | | | | | | | | | | | |
| District | **Crop farming(%)** | | | | **Livestock farming(%)** | | | | **Employment (%)** | | | |
|  | **2009/10** | **2013** | **2015** | **p for time** | **2009/10** | **2013** | **2015** | **p for time** | **2009/10** | **2013** | **2015** | **P for time** |
| Dolpa^a^ | 48.4 | 72.3 | 31.4 | <0.001 | 5.2 | 1.2 | 9.7 | <0.001 | 26.6 | 14.1 | 24.6 | <0.001 |
| Jumla^a^ | 44.3 | 48.5 | 27.8 | <0.001 | 2.7 | 5.1 | 4.9 | 0.049 | 44.4 | 31.2 | 35.5 | <0.01 |
| Mugu^a^ | 53.2 | 68.0 | 42.9 | <0.001 | 3.5 | 3.9 | 11.5 | <0.001 | 23.8 | 24.0 | 38.3 | <0.001 |
| Humla^a^ | 35.9 | 49.1 | 43.4 | <0.001 | 6.7 | 5.3 | 9.1 | 0.030 | 52.5 | 42.0 | 40.2 | <0.001 |
| Kalikot^a^ | 47.7 | 60.8 | 23.3 | <0.001 | 0.8 | 1.7 | 6.8 | <0.001 | 35.2 | 25.6 | 34.8 | <0.01 |
| Karnali Zone^b^ | 45.9 | 59.7 | 33.6 | <0.001 | 3.8 | 3.4 | 8.6 | <0.001 | 36.5 | 27.4 | 33.9 | <0.001 |

^a^ Two-level GLMMs with binominal link were used to adjust for clustering of children within wards. ^b^ Three-level GLMMs with binominal link were used to adjust for clustering within districts and clustering of children within wards. .
